# Synthesis and solid-state characterisation of 4-substituted methylidene oxindoles

**DOI:** 10.1186/1752-153X-7-182

**Published:** 2013-12-20

**Authors:** Graham J Tizzard, Simon J Coles, Mark Edwards, Romanus Oforbike Onyeabo, Mark Allen, John Spencer

**Affiliations:** 1 UK National Crystallography Service, School of Chemistry, University of Southampton, SO17 1BJ, Southampton, UK; 2 The School of Science, University of Greenwich, Medway Campus, Central Avenue, ME4 4TB, Chatham Maritime, Kent, UK; 3 Department of Chemistry, School of Life Sciences, University of Sussex, Falmer, BN1 9NQ, Brighton, UK

## Abstract

**Background:**

4-substituted methylidene oxindoles are pharmacologically important. Detailed analysis and comparison of all the interactions present in crystal structures is necessary to understand how these structures arise. The XPac procedure allows comparison of complete crystal structures of related families of compounds to identify assemblies that are mainly the result of close-packing as well as networks of directed interactions.

**Results:**

Five 4-substituted methylidene oxindoles have been synthesized by the Knoevenagel condensation of oxindole with para-substituted aromatic aldehydes and were characterized in the solid state by x-ray crystallography. Hence, the structures of (3E)-3-(4-Bromobenzylidene)-1,3-dihydro-2H-indol-2-one, 3a, (3E)-3-(4-Chlorobenzylidene)-1,3-dihydro-2H-indol-2-one, 3b, (3E)-3-(4-Methoxybenzylidene)-1,3-dihydro-2H-indol-2-one, 3c, (3E)-3-(4-Methylbenzylidene)-1,3-dihydro-2H-indol-2-one, 3d and (3E)-3-(4-Nitrobenzylidene)-1,3-dihydro-2H-indol-2-one, 3e, were elucidated using single crystal X-ray crystallography.

**Conclusions:**

A hydrogen bonded dimer molecular assembly or supramolecular construct was identified in all the crystal structures examined along with a further four 1D supramolecular constructs which were common to at least two of the family of structures studied. The 1D supramolecular constructs indicate that once the obvious strong interaction is satisfied to form hydrogen bonded dimer it is the conventionally weaker interactions, such as steric bulk and edge-to-face interactions which compete to influence the final structure formation.

## Introduction

Oxindoles are important scaffolds in medicinal chemistry and synthetic and structural studies on this important class of heterocycles are extremely welcome. We have recently described the synthesis of a range of oxindoles [1] and biological studies on metal-substituted oxindole complexes have revealed impressive kinase inhibition [2]. Supported by solid-state analysis, we have been able to establish structure-activity relationships in the oxindoles reported in these studies. The pharmacological importance of 4-substituted methylidene oxindoles cannot be overemphasized. Many have anticancer properties and inhibit vascular endothelial growth factor receptor-2 (VEGFR-2) and platelet-derived growth factor receptor (PDGFR), linked with angiogenesis [3,4].

The burgeoning scientific interest in the study of polymorphism, crystal engineering and crystal structure prediction has resulted in the need for systematic analysis protocols to enable the comparison of different crystal structures. In 1998 Nangia and Desiraju [5] argued that a full understanding of crystal structure and crystal engineering requires a comparison of the entire molecule and all interactions in the crystalline state. The analysis of crystal structures for similarities and differences is one of the key issues facing structural chemists today and to that end a number of methods have been developed in recent years to compare crystal structures [6-11]. Many of these have concentrated on the comparison of subsets of structures i.e. comparing polymorphs of a single compound, or the analysis of directed interactions such as hydrogen bonding. However, crystal structures are assembled by the interplay of a number of forces and thus these methods compare only a subset of the interactions contained within crystal structures. To be of more general utility, an analysis method should be flexible enough to identify components of a structure that may reflect the influence of the more diffuse interactions and thus be able to identify assemblies that are mainly the result of close-packing as well as networks of directed interactions. It should also be able to compare crystal structures of different molecular species to allow the systematic investigation of related families of structures, thus allowing the investigation of the effects of systematic substituent variation. With these points in mind the XPac [12,13] procedure has been developed in our laboratory, the use of which is explained below.

Herein we report the solid-state study of five 4-substituted benzylidene oxindoles and the XPac analyses of these along with three previously published structures.

## Experimental

### Synthesis

**Scheme 1:**
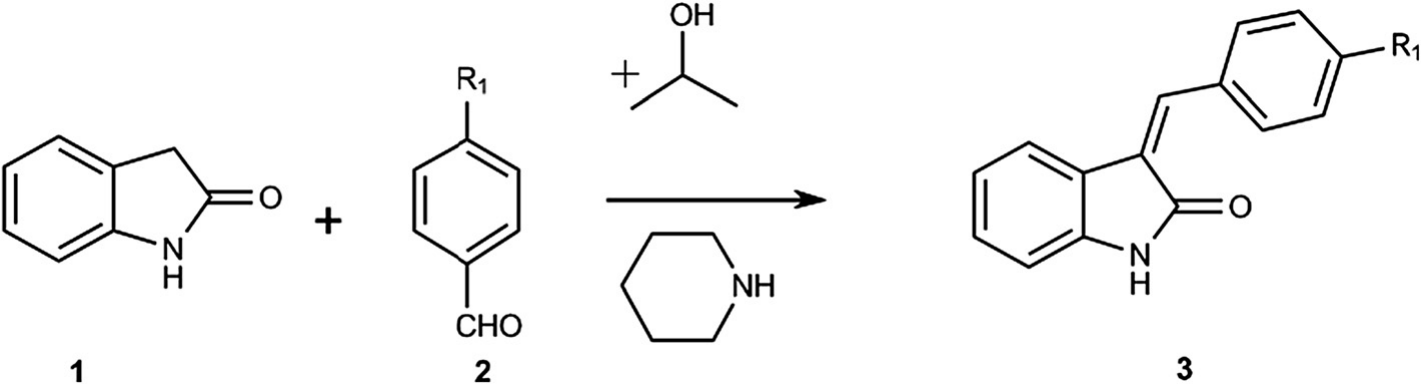
Reaction scheme for 3-substituted methylidene oxindoles, 3a-e.

The general reaction scheme outlined below (Scheme 1) was followed, with specific details of the synthesis procedures of all the derivatives of 3 available at http://poc.labtrove.soton.ac.uk/synth_methyl_oxin/group/Condensation%20Products.

Where R_1_ = Br(3a), InChIKey = INAOSTJXLBNFMV-UKTHLTGXSA-N

Cl (3b) InChIKey = CIXKMQYKNWKSNZ-UKTHLTGXSA-N.

OMe (3c) InChIKey = SOHLANGNFXOOEF-GXDHUFHOSA-N.

Me (3d) InChIKey = JEZSEMYAZZHQIB-GXDHUFHOSA-N.

NO_2_ (3e) InChIKey = WPZSQSSOBHCYOZ-UKTHLTGXSA-N.

General: A solution of 1 (1.37g, 10mmol) and 2 (10mmol) in propan-2-ol (10ml, 100mmol) and piperidine (0.5ml, 5mmol) was refluxed at 100°C for 3h. On cooling the resultant solid was filtered, dried under reduced pressure and then purified by sublimation to give the product. Full details of experimental processes and observations of the synthesis of 3a-e are available at http://poc.labtrove.soton.ac.uk/synth_methyl_oxin/group/Experimental%20Procedure.

3a: doi:10.5258/poc/lt/r/1

Yellow solid; yield: 1.62g, 5.40mmol, 54%; mp: 192.5°C; IR (ν_max_, cm^-1^) 1700 (C=O stretch); ^1^H (CDCl_3_): 6.85-6.90 (2H, m), 7.26-7.29 (2H, m), 7.59-7.62 (4H, m), 7.72 (1H, s), 8.28 (1H, brs). ^13^C (CDCl_3_): 110.2, 121.4, 121.8, 122.0, 123.0, 123.8, 127.9, 130.1, 130.2, 130.8, 131.5, 131.9, 133.7, 135.9, 169.7; ESIMS (positive mode) (m/z): 300.1 [M+H]^+^, 302.1 [M+H]^+^.

3b: doi:10.5258/poc/lt/r/2

Yellow solid; yield: 1.93g, 7.56mmol, 76%; mp: 181.5°C; IR (ν_max_, cm^-1^) 1700 (C=O stretch); ^1^H (CDCl_3_): 6.89-6.92 (2H, m), 7.25-7.28 (2H, m), 7.46 (2H, m), 7.58-7.62 (2H, m), 7.75 (1H, s), 8.53 (1H, brs). ^13^C (CDCl_3_): 110.2, 110.4, 121.4, 121.8, 122.0, 122.9, 128.0, 128.9, 130.1, 130.2, 130.6, 133.2, 135.5, 135.8, 169.9; ESIMS (positive mode) (m/z): 256.2 [M+H]^+^, 258.2 [M+H]^+^.

3c: doi:10.5258/poc/lt/r/3

Yellow solid; yield: 1.62g, 6.45mmol, 65%; mp: 152.2°C; IR (ν_max_, cm^-1^) 1696 (C=O stretch); ^1^H (CDCl_3_): 3.89 (3H, s), 6.89-6.92 (2H, m), 6.98-7.01 (2H, m), 7.25 (1H, m), 7.65-7.68 (2H, m), 7.75 (1H, m), 7.80 (1H, s), 8.51 (1H, brs). ^13^C (CDCl_3_): 55.4, 110.1, 114.0, 114.1, 121.7, 122.0, 122.7, 125.6, 127.1, 129.4, 131.5, 137.7, 141.3, 160.9, 170.5; ESIMS (positive mode) (m/z): 252.2 [M+H]^+^.

3d: doi:10.5258/poc/lt/r/4

Yellow solid; yield: 1.61g, 6.84mmol, 68%; mp: 187.0°C; IR (ν_max_, cm^-1^) 1690 (C=O stretch); ^1^H (DMSO-_d6_): 2.43 (3H, s), 6.87-6.92 (2H, m), 7.25-7.29 (2H, m), 7.52 (2H, d, J=7.9), 7.60 (2H, d, J= 7.9), 7.82 (1H, s), 8.23 (1H, s). ^13^C (CDCl_3_): 21.5, 110.2, 121.7, 121.8, 122.9, 123.9, 129.1, 129.4, 129.5, 129.6, 131.9, 132.2, 137.8, 140.1, 141.5, 170.7; ESIMS (positive mode) (m/z): 236.3 [M+H]^+^, 471.5 [2M+H]^+^.

3e: doi:10.5258/poc/lt/r/5

Red solid; yield: 1.80g, 6.76mmoml, 68%; mp: 247.0°C; IR (ν_max_, cm^-1^) 1697 (C=O stretch); ^1^H (dmso-d_6_): 6.81-6.91 (2H, m), 7.21 (1H, pseudo tr, J=7.5), 7.39 (1H, ps tr, J=7.5), 7.68 (1H, s), 7.80 (2H, d, J= 8.6), 8.34 (2H, d, J=8.6), 10.70 (1H, brs). ^13^C (dmso-d6): 110.7, 121.5, 122.9, 123.8, 129.9, 130.9, 132.4, 141.9, 143.3, 147.7, 161.1, 169.1 (some quaternaries missing, poor solubility); ESIMS (positive mode) (m/z): 265.2 [M+H]^+^.

Full details of characterisation techniques and data for 3a-e are available at http://poc.labtrove.soton.ac.uk/synth_methyl_oxin/group/Analytical%20Procedures and http://poc.labtrove.soton.ac.uk/synth_methyl_oxin/group/Spectroscopic%20Data.

### Single Crystal X-ray crystallography

Single-crystal X-ray diffraction analyses were performed using a Bruker APEXII CCD diffractometer mounted at the window of a Bruker FR591 rotating anode (MoKα = 0.71073 Å) and equipped with an Oxford Cryosystems cryostream device. Data were processed using the Collect package and unit cell parameters were refined against all data. An empirical absorption correction was carried out using SADABS. The structures were solved by direct methods using SHELXS-97 and refined on F_o_^2^ by full-matrix least-squares refinements using SHELXL-97. All non-hydrogen atoms were refined with anisotropic displacement parameters. All hydrogen atoms were added at calculated positions and refined using a riding model with isotropic displacement parameters based on the equivalent isotropic displacement parameter (Ueq) of the parent atom. Figures were produced using OLEX2. A summary of the crystallographic data is shown below (Table 1) and full details of the crystallographic experiment for each of the compounds are available at:

3a doi:10.5258/ecrystals/1505

3b doi:10.5258/ecrystals/1326

3c doi:10.5258/ecrystals/1327

3d doi:10.5258/ecrystals/1324

3e doi:10.5258/ecrystals/1325

**Table 1:** Crystallographic data for 4-substituted methylidene oxindoles

	**3a**	**3b**	**3c**	**3d**	**3e**
Empirical Formula	C_15_H_12_BrNO_2_	C_15_H_12_ClNO_2_	C_16_H_13_NO_2_	C_16_H_13_NO	C_15_H_10_N_2_O_3_
Formula Weight	318.17	273.71	251.27	235.27	266.25
T/K	120(2)	120(2)	120(2)	120(2)	120(2)
Crystal System	Monoclinic	Monoclinic	Triclinic	Triclinic	Monoclinic
Space Group	*C*2/*c*	*C*2/*c*	*P*−1	*P*−1	*P*21/*c*
*a*/Å	19.623(3)	19.6553(12)	4.1302(2)	8.1168(4)	9.9484(11)
*b*/Å	4.0710(5)	4.0406(2)	12.9513(9)	9.2556(4)	7.9134(10)
*c*/Å	32.979(4)	32.653(2)	13.0829(9)	9.3927(3)	16.013(2)
α/°	90	90	62.708(3)	62.290(2)	90
β/°	101.698(3)	101. 378(2)	86.495(4)	80.933(3)	104.340(8)
γ/°	90	90	86.546(4)	72.180(2)	90
V/Å^3^	2579.8(6)	2542.3(3)	620.35(7)	594.64(4)	1221.4(3)
*D*_calcd_/g cm^-3^	1.638	1.430	1.345	1.314	1.448
Independent reflections	2888	2890	2830	2701	2062
*R*_*int*_	0.0715	0.0578	0.0592	0.0506	0.0526
Data/restraints/parameters	2888/7/181	2890/7/181	2830/0/173	2701/0/165	2062/0/181
Absorption coefficient/mm^-1^	3.183	0.297	0.089	0.082	0.103
*R*_1_/w*R*_2_ (observed data: *F*^2^ > 2σ(*F*^2^))	0.0785/0.1445	0.0470/0.1209	0.0877/0.1398	0.0647/0.1240	0.0623/0.1246
*R*1/w*R*_2_ (all data)	0.1198/0.1671	0.0657/0.1320	0.1415/0.1633	0.0924/0.1419	0.1026/0.1497

## Results and discussion

### Discussion of crystal structures

**Figure 1:**
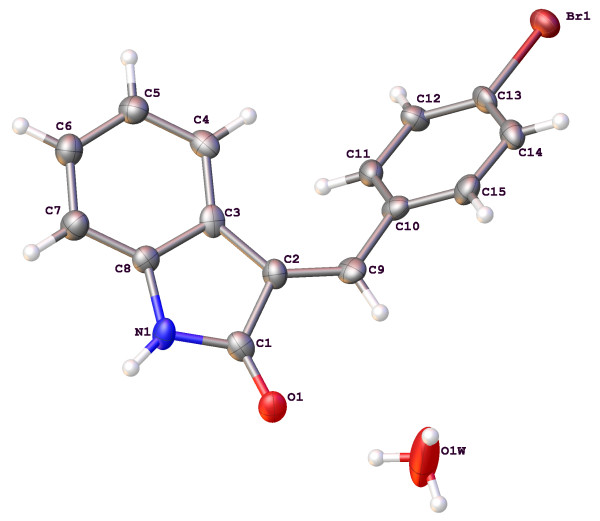
Molecular structure and atomic numbering scheme of compound 3a.

**Figure 2:**
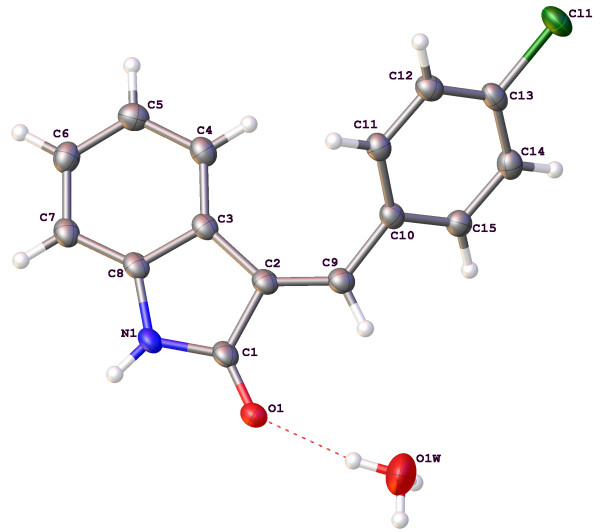
Molecular structure and atomic numbering scheme of compound 3b.

**Figure 3:**
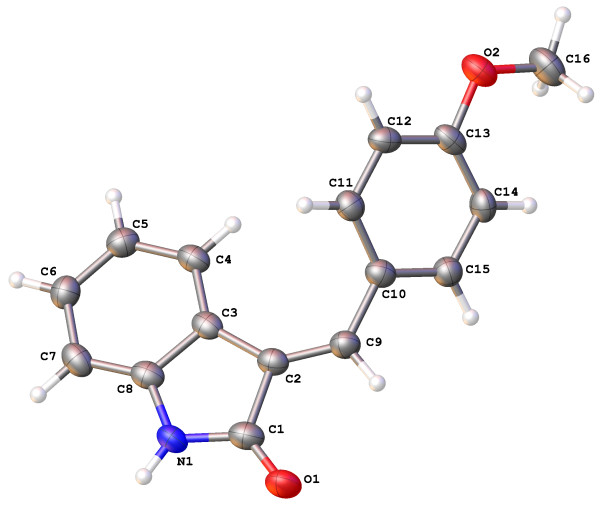
Molecular structure and atomic numbering scheme of compound 3c.

**Figure 4:**
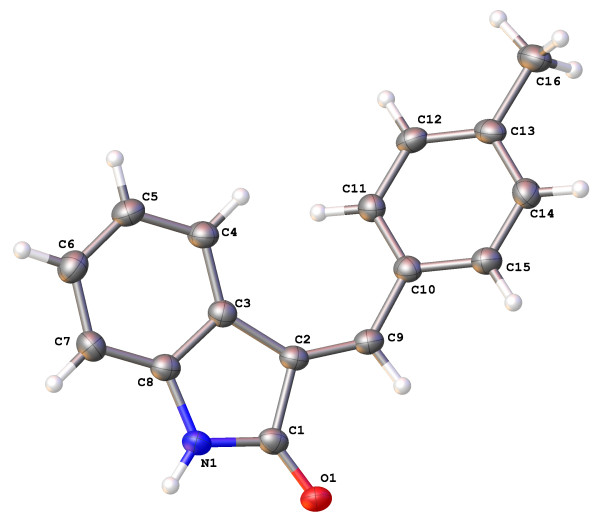
Molecular structure and atomic numbering scheme of compound 3d.

**Figure 5:**
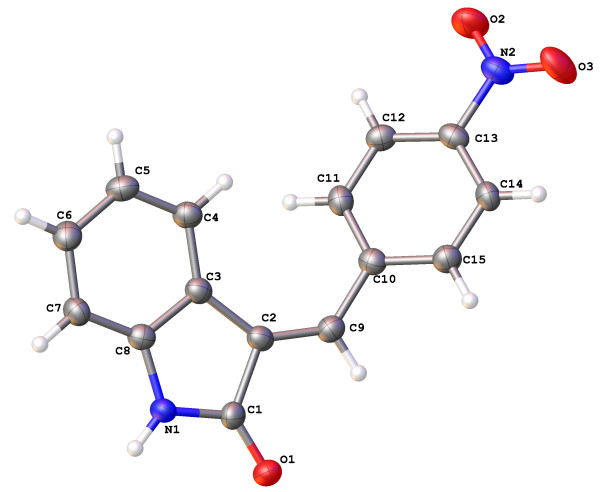
Molecular structure and atomic numbering scheme of compound 3e.

The molecular structures and numbering schemes for compounds 3a-e are depicted in Figures 1, 2, 3, 4 and 5 below and confirm the expected substitution patterns for this homologous series. The structures of 3a and 3b are isostructural and both contain channels of hydrogen bonded water molecules running parallel to the b-axis. These channels contain water molecules in two alternate orientations so that in each case one H-atom forms a hydrogen bond with the carbonyl O-atom of the oxindole (3a, O^…^O distance = 2.815(9)Å, O-H^…^O angle = 151(13)°; 3b, O^…^O distance = 2.813(3)Å, O-H^…^O angle = 171(5)°). Thus in these structures, the carbonyl O-atom acts as a H-bond acceptor to two H-bond donors, the water molecules and the oxindole amine N-H. The remaining H-atom of the water molecule H-bonds to an adjacent water molecule in the structure (3a, O^…^O distance = 2.68(3)-2.72(2)Å, O-H^…^O angle = 154(8)-167(12)°; 3b, O^…^O distance = 2.713(8)-2.718(6)Å, O-H^…^O angle = 158(7)°) to form the water channel. The conformation of the molecular structures of these compounds is predominantly defined by rotation about a single methylene group (C9) and all five structures exhibit approximately the same shape (oxindole-benzyl interplanar angle range: 36.11-49.56°). This observation indicates that the different substituents in the para position studied as part of this work do not significantly affect molecular conformation.

### Comparison of crystal structures

The different substituents in this series do however affect the potential for the structure-defining formation of intermolecular interactions as evidenced by the different crystal structures exhibited by these molecules. Therefore a study using the XPac approach, an algorithm developed to compare sets of complete crystal structures, has been conducted. With this technique, the comparison of structures is based purely on relative geometric conformations and positions of molecules without bias from perceived chemical effects such as H-bonding or other directional intermolecular interactions. The comparison of a group of crystal structures is carried out in a pairwise fashion and is accomplished by generating a set of neighbouring molecules, analogous to a coordination sphere, around a central molecule from the symmetry operations of the space groups of each structure. Parameter lists are then generated and compared for each crystal structure based on angular, planar and distance relationships between the kernel molecule (at the centre of the cluster) and each molecule in the cluster (the surrounding neighbouring molecules). Any matches (within user defined tolerances) of these sets of parameters equates to matches in the positions of molecules in the clusters for each of the crystal structures (the kernels always match of course) and depending on the number of matches the dimensionality of the similarity may be derived. Thus crystal structures with common discrete assemblies e.g. dimers display 0D similarity, those with common rows or stacks of molecules display 1D similarity, structures in which layers of molecules match display 2D similarity and structures which are isostructural display 3D similarity. These common structural motifs between structures are termed Supramolecular Constructs (SCs).

The set of five structures detailed above, along with a further three previously characterized 3-substituted methylidene oxindoles: (3E)-3-(4-Ethylbenzylidene)-1,3-dihydro-2H-indol-2-one [14], 3f, (3Z)-3-(Benzylidene)-1,3-dihydro-2H-indol-2-one [15], 4a and (3Z)-3-(4-Chlorobenzylidene)-1,3-dihydro-2H-indol-2-one [16], 4b, obtained from the Cambridge Structural Database (CSD) [17] were compared using the XPac algorithm and the following SCs (Table 2) were identified.

**Table 2:** Similarity relationships amongst oxindoles studied (D = dimensionality, # = number of structures)

**SC**	**D**	**Description**	**Figs.**	**#**	**Base vector(s)**	**Dependencies**
A01	0	N-H…O H-bonded oxindole dimer	7	7		Primary SC
A11	1	A01 dimer ‘stack’	8	3	t1	A11→A01
A12	1	A01 dimer ‘sheared tape’	9	2	t2	A12→A01
A13	1	A01 dimer ‘tape’	10	2	t3	A13→A01
A31	3	Isostructural set		2	t1, t4, t5	A31→A11
B11	1	ring ‘edge to face’ tape	11	2	t6	Primary SC

**Figure 6:**
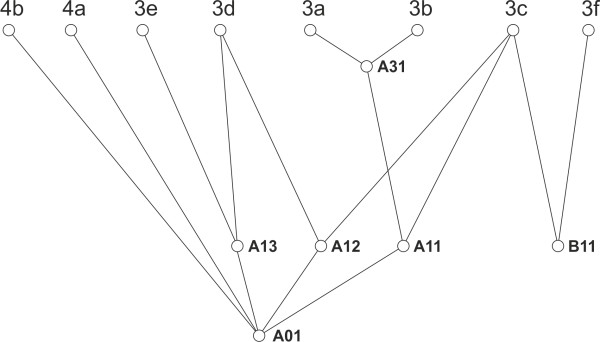
The relationship and notation of the common repeat motifs (SCs) in the crystal structures of compounds 3a-f and 4a-b.

In order to discuss the similarity relationships between crystal structures, a method for their graphical representation needs to be described. Each SC provides a connection between at least two crystal structures and a SC may itself be derived from one or more SCs (its sub SCs). Thus in the structure relationship diagram below (Figure 6), each element within a family i.e. crystal structures and SCs is represented by a node and each edge connecting the nodes represents a dependency between these elements. There is a strict vertical hierarchy such that for connected nodes the higher node is a subgroup of the lower node, whilst the horizontal arrangement is arbitrary and simply arranged to provide the least number of crossing lines for ease of readability. The order of elements from bottom to top are 0D SCs < 1D SCs < 2D SCs < 3D SCs < crystal structures, thus to find all the crystal structures that contain a particular SC, all of the branches radiating *upwards* from its node are followed to the crystal structure level. To find a common SC of two crystal structures the branches radiating *downwards* from the nodes representing the crystal structures are followed until they meet at a common node.

**Figure 7:**
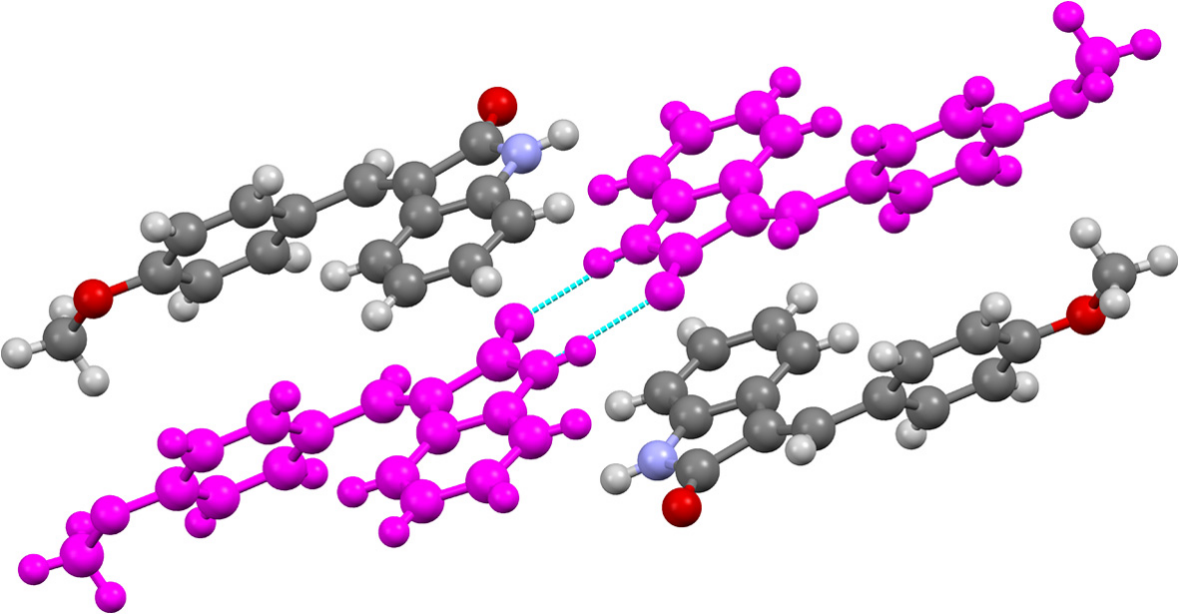
**Crystal structure of 3c viewed along bc-axis with SC A01 (N-H…O H-bonded dimer) highlighted in pink.** H-bonds are shown in light blue.

The XPac analysis of the eight crystal structures reveals some interesting packing features. As one might predict, the dominant packing feature of this set of structures is the N-H^…^O dimer formed by the oxindole moieties of the structures, SC A01 (Figure 7). The geometries of the dimers are unremarkable with D^…^A distances of 2.844(7)-2.863(4)Å and D-H^…^A angles between 167-169° for the Z-conformers and between 2.867(3)-2.904(3)Å and 172-177° for the E-conformers. The larger intermolecular distances and angles of the E-conformers compared with the Z-conformers can be accounted for by steric effects. The dimer motif is robust enough so that the presence of water molecules in the two hydrate structures does not disrupt its formation. The dimer is present in all of the oxindole structures investigated except 3f which forms a catamer structure with a D^…^A distance of 2.850(4)Å and a D-H^…^A angle of 167°. Also, this is the only structure which exhibits disorder with the benzylidene ring of the molecule adopting two conformations.

**Figure 8:**
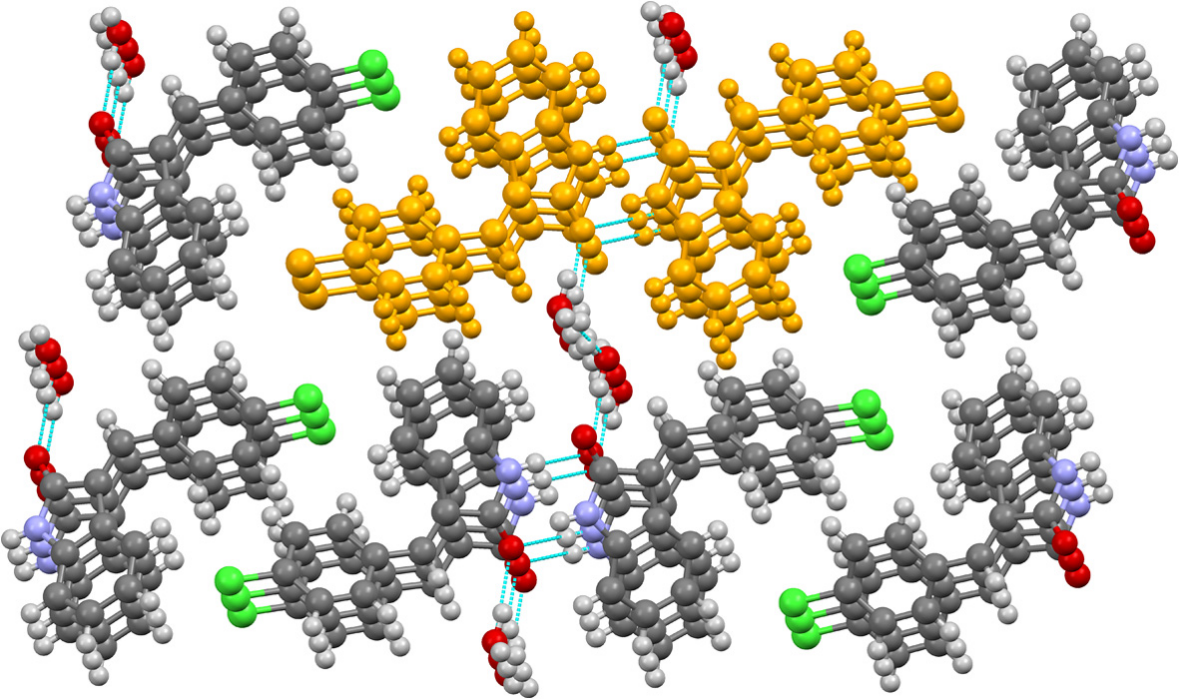
**Crystal structure of 3b viewed approximately along the b-axis with SC A11 (H-bonded dimer stack) highlighted in gold.** H-bonds are shown in light blue.

SC A11 (Figure 8) is a 1D dimer stack with a translation vector t1 (4.041-4.130Å). It is present in three of the eight oxindole structures investigated: 3a, 3b and 3c. 3a and 3b are isostructural, related by 3D SC A31 and thus this is a subgroup of SC A11. In the structures of 3a and 3b SC A11 propagates along the b-axis whilst in 3c it propagates along the a-axis. Interestingly, there are no direct close contacts less than the sum of the van der Waals (vdW) radii between the dimers comprising SC A11 in any of the structures in which it occurs. However in all the structures studied, close contacts less than the sum of the vdW radii from constituent dimers of the SC to neighboring molecules outside of the SC are observed suggesting that SC A11 arises purely as a result of simple close-packing considerations in these structures.

**Figure 9:**
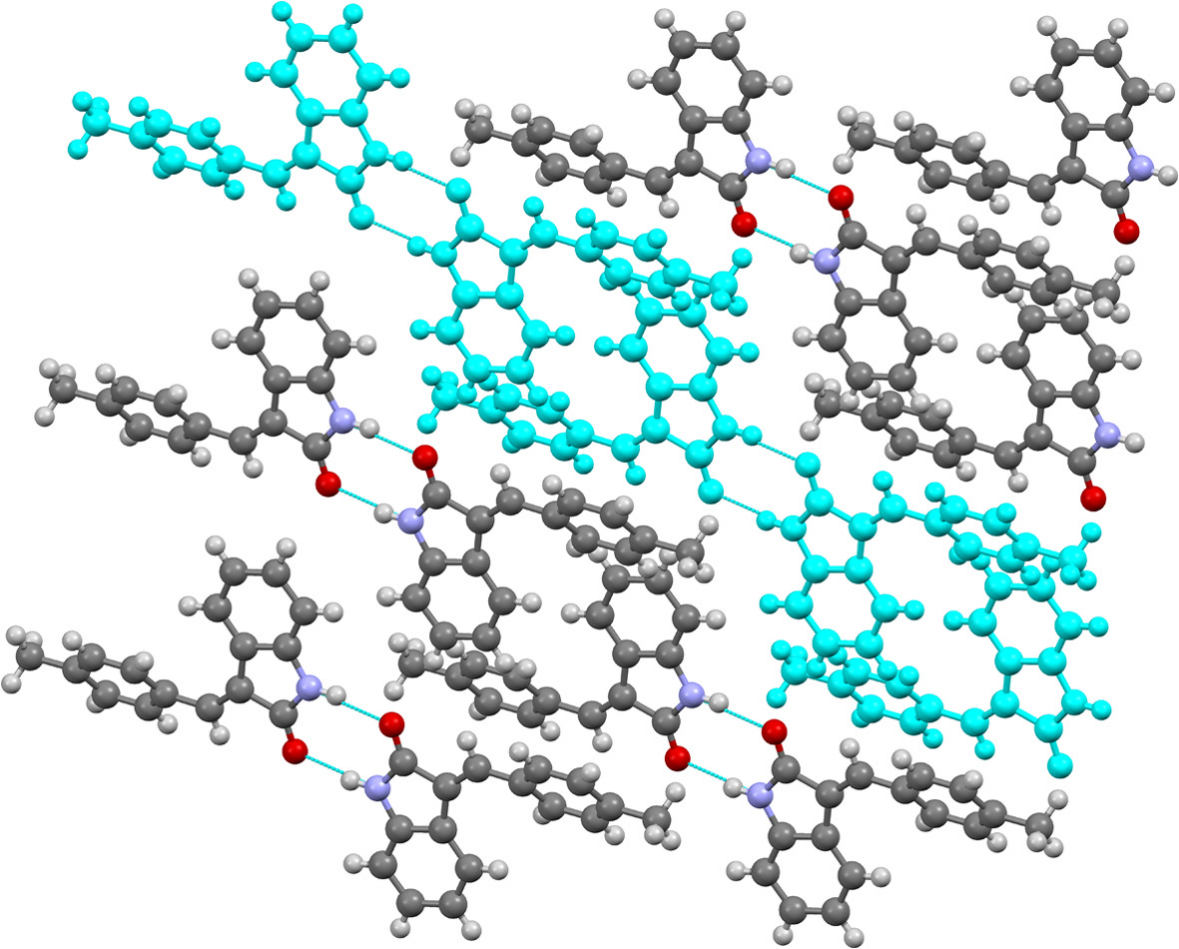
**Crystal structure of 3d viewed approximately along the ac-axis with SC A12 (H-bonded dimer tape) highlighted in light blue.** H-bonds are shown in light blue.

SC A12 (Figure 9) is best described as a 1D dimer ‘sheared’ tape and is present in the structures of 3c and 3d with a translation vector t2 (13.355-13.449Å). Both 3c and 3d display layer structures with dimer layers parallel to the 120 and 201 planes respectively and SC A12 comprises adjacent dimers from adjacent layers, hence the ‘sheared’ tape description. In the structure of 3c SC A12 propagates along the a-b direction whilst in 3d it propagates along the -a-bc direction. The constituent dimers of SC A12 in 3c exhibit a double close contact less than the sum of the vdW radii between C6 of the oxindole moiety and H12 (of the benzylidene moiety (2.769Å). However, there are no close contacts less than the sum of the vdW radii between the constituent dimers of SC A12 in 3d and so these are not expected to be integral to the SC’s formation.

**Figure 10:**
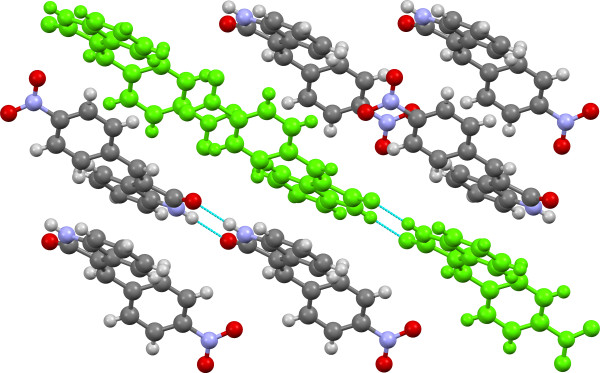
**Crystal structure of 3e viewed approximately along the c-axis with SC A13 (H-bonded dimer tape) highlighted in green.** H-bonds are shown in light blue.

SC A13 (Figure 10) is a 1D dimer tape and is present in the structures of 3d and 3e with a translation vector t4 (18.457-18.694Å). As previously mentioned, 3d forms a simple layer structure and instances of SC A13 propagate along these layers. The structure of 3e is more complex with adjacent stacks of dimer tapes (SC A13 stacks) rotated 62.7° with respect to each other. Once again one of the structures displaying this SC (3d) also displays short contacts below the sum of the vdW radii between constituent dimers of the SC. These however are very minor, comprising a double contact between C16 of the methyl substituent and H4 of the oxindole (2.899Å) and this along with the fact that the structure of 3e displays no short contacts below the sum of the vdW radii between constituent dimers of the SC suggest that these are not SC forming interactions.

**Figure 11:**
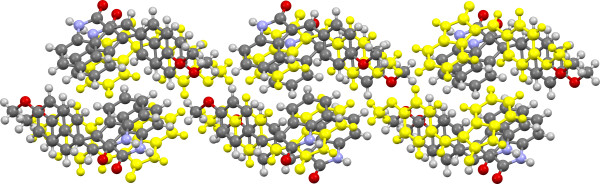
Crystal structure of 3c viewed approximately along the a-axis with SC B11 (ring ‘edge to face’ tape) highlighted in yellow.

SC B11 (Figure 11) is the only SC amongst this group of structures which is not based on the H-bonded dimer, SC A01. The SC consists of a repeating double-molecule motif forming a 1D tape as shown below. It is displayed in the structures of 3c and 3f where it propagates along a-c axis and a-b axis respectively. In common with structure displaying previously outlined SCs, the structure of 3c displays only a single, minor short contact less than the sum of the vdW amongst constituent molecules of the SC between C16 of the methoxy substituent and H7 of the indole moiety (2.847Å). No such contacts are observed amongst the constituent molecules of the SC in the structure of 3f suggesting that this SC is not directly structure forming.

## Conclusions

As can be seen from the analysis above, the only SC which would conventionally be seen as containing structure forming intermolecular interactions is SC A01, the H-bonded dimer. The other SCs, apart from B11, are all based on the A01 dimer as a building block. However, the other SCs do not display any further significant relationships that could be considered to be classical strong hydrogen bonds. Nevertheless, each of these SCs have been identified in at least two different crystal structures suggesting that they are robust enough to have arisen under at least two different sets of crystallisation conditions. Despite possessing a strong and constrained structure-forming motif in the oxindole moiety and a relatively simple substitution pattern in the compound family a variety of SCs are formed. This indicates that once the obvious strong interaction is satisfied to form the primary SC it is the conventionally weaker interactions, such as steric bulk and edge-to-face exhibited here, that compete to influence the final structure formation.

## Competing interests

The authors declare that they have no competing interests.

## Authors’ contributions

ME, ROO, MA, JS synthesized and characterized 4-substituted methylidene oxindoles. GJT carried out crystallographic analysis and undertook XPac analysis. GJT, SJC prepared the initial and final drafts of the manuscript. All authors read and approved final manuscript.

## Supplementary Material

Additional file 1 **DeepZoom: 4-methylidene oxindole synthesis and characterization.** Microsoft Silverlight plug-in is required to view this. **Use + and - buttons or scroll wheel to zoom in and out of image to view individual spectra.** Use home button to reset view to full image. Click table cells to follow link to relevant section of electronic lab notebook for full supporting data (spectral assignments etc). Click here for file
