# Supplementary figures and images for: Synthesis and solid-state characterisation of 4-substituted methylidene oxindoles (part 1 of 2)
Source: Chem Cent J. 2013 Dec 20;7:182. doi: 10.1186/1752-153X-7-182 (PMC3878136; doi:10.1186/1752-153X-7-182)

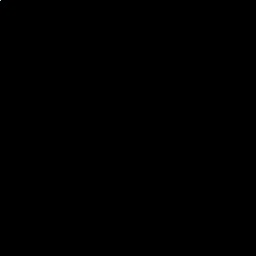

Supplement: Additional file 1 — DeepZoom: 4-methylidene oxindole synthesis and characterization. Microsoft Silverlight plug-in is required to view this. Use + and - buttons or scroll wheel to zoom in and out of image to view individual spectra. Use home button to reset view to full image. Click table cells to follow link to relevant section of electronic lab notebook for full supporting data (spectral assignments etc). [file 1752-153X-7-182-S1.zip › GeneratedImages/dzc_output_files/0/0_0.jpg]

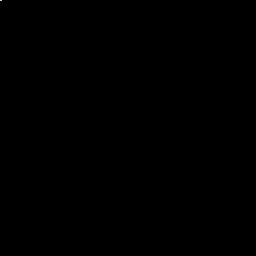

Supplement: Additional file 1 — DeepZoom: 4-methylidene oxindole synthesis and characterization. Microsoft Silverlight plug-in is required to view this. Use + and - buttons or scroll wheel to zoom in and out of image to view individual spectra. Use home button to reset view to full image. Click table cells to follow link to relevant section of electronic lab notebook for full supporting data (spectral assignments etc). [file 1752-153X-7-182-S1.zip › GeneratedImages/dzc_output_files/1/0_0.jpg]

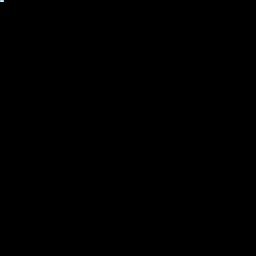

Supplement: Additional file 1 — DeepZoom: 4-methylidene oxindole synthesis and characterization. Microsoft Silverlight plug-in is required to view this. Use + and - buttons or scroll wheel to zoom in and out of image to view individual spectra. Use home button to reset view to full image. Click table cells to follow link to relevant section of electronic lab notebook for full supporting data (spectral assignments etc). [file 1752-153X-7-182-S1.zip › GeneratedImages/dzc_output_files/2/0_0.jpg]

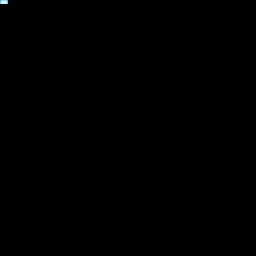

Supplement: Additional file 1 — DeepZoom: 4-methylidene oxindole synthesis and characterization. Microsoft Silverlight plug-in is required to view this. Use + and - buttons or scroll wheel to zoom in and out of image to view individual spectra. Use home button to reset view to full image. Click table cells to follow link to relevant section of electronic lab notebook for full supporting data (spectral assignments etc). [file 1752-153X-7-182-S1.zip › GeneratedImages/dzc_output_files/3/0_0.jpg]

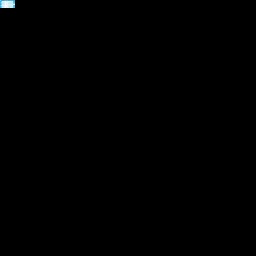

Supplement: Additional file 1 — DeepZoom: 4-methylidene oxindole synthesis and characterization. Microsoft Silverlight plug-in is required to view this. Use + and - buttons or scroll wheel to zoom in and out of image to view individual spectra. Use home button to reset view to full image. Click table cells to follow link to relevant section of electronic lab notebook for full supporting data (spectral assignments etc). [file 1752-153X-7-182-S1.zip › GeneratedImages/dzc_output_files/4/0_0.jpg]

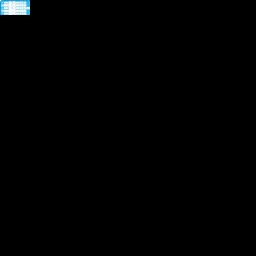

Supplement: Additional file 1 — DeepZoom: 4-methylidene oxindole synthesis and characterization. Microsoft Silverlight plug-in is required to view this. Use + and - buttons or scroll wheel to zoom in and out of image to view individual spectra. Use home button to reset view to full image. Click table cells to follow link to relevant section of electronic lab notebook for full supporting data (spectral assignments etc). [file 1752-153X-7-182-S1.zip › GeneratedImages/dzc_output_files/5/0_0.jpg]

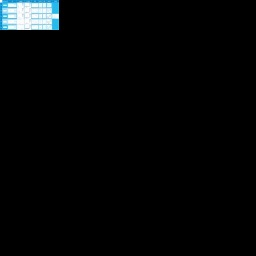

Supplement: Additional file 1 — DeepZoom: 4-methylidene oxindole synthesis and characterization. Microsoft Silverlight plug-in is required to view this. Use + and - buttons or scroll wheel to zoom in and out of image to view individual spectra. Use home button to reset view to full image. Click table cells to follow link to relevant section of electronic lab notebook for full supporting data (spectral assignments etc). [file 1752-153X-7-182-S1.zip › GeneratedImages/dzc_output_files/6/0_0.jpg]

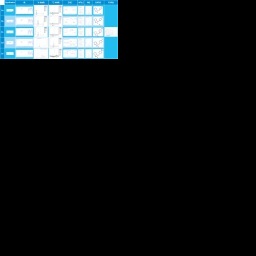

Supplement: Additional file 1 — DeepZoom: 4-methylidene oxindole synthesis and characterization. Microsoft Silverlight plug-in is required to view this. Use + and - buttons or scroll wheel to zoom in and out of image to view individual spectra. Use home button to reset view to full image. Click table cells to follow link to relevant section of electronic lab notebook for full supporting data (spectral assignments etc). [file 1752-153X-7-182-S1.zip › GeneratedImages/dzc_output_files/7/0_0.jpg]

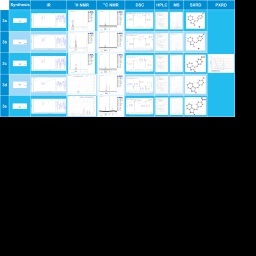

Supplement: Additional file 1 — DeepZoom: 4-methylidene oxindole synthesis and characterization. Microsoft Silverlight plug-in is required to view this. Use + and - buttons or scroll wheel to zoom in and out of image to view individual spectra. Use home button to reset view to full image. Click table cells to follow link to relevant section of electronic lab notebook for full supporting data (spectral assignments etc). [file 1752-153X-7-182-S1.zip › GeneratedImages/dzc_output_files/8/0_0.jpg]

Supplement: Additional file 1 — DeepZoom: 4-methylidene oxindole synthesis and characterization. Microsoft Silverlight plug-in is required to view this. Use + and - buttons or scroll wheel to zoom in and out of image to view individual spectra. Use home button to reset view to full image. Click table cells to follow link to relevant section of electronic lab notebook for full supporting data (spectral assignments etc). [file 1752-153X-7-182-S1.zip › GeneratedImages/dzc_output_images/gjtccjesi160113_files/0/0_0.jpg]

Supplement: Additional file 1 — DeepZoom: 4-methylidene oxindole synthesis and characterization. Microsoft Silverlight plug-in is required to view this. Use + and - buttons or scroll wheel to zoom in and out of image to view individual spectra. Use home button to reset view to full image. Click table cells to follow link to relevant section of electronic lab notebook for full supporting data (spectral assignments etc). [file 1752-153X-7-182-S1.zip › GeneratedImages/dzc_output_images/gjtccjesi160113_files/1/0_0.jpg]

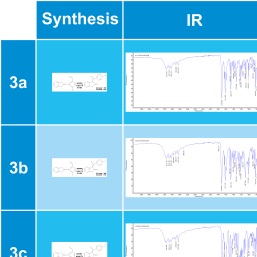

Supplement: Additional file 1 — DeepZoom: 4-methylidene oxindole synthesis and characterization. Microsoft Silverlight plug-in is required to view this. Use + and - buttons or scroll wheel to zoom in and out of image to view individual spectra. Use home button to reset view to full image. Click table cells to follow link to relevant section of electronic lab notebook for full supporting data (spectral assignments etc). [file 1752-153X-7-182-S1.zip › GeneratedImages/dzc_output_images/gjtccjesi160113_files/10/0_0.jpg]

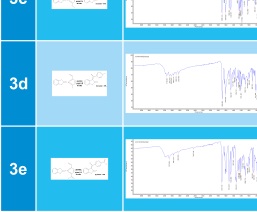

Supplement: Additional file 1 — DeepZoom: 4-methylidene oxindole synthesis and characterization. Microsoft Silverlight plug-in is required to view this. Use + and - buttons or scroll wheel to zoom in and out of image to view individual spectra. Use home button to reset view to full image. Click table cells to follow link to relevant section of electronic lab notebook for full supporting data (spectral assignments etc). [file 1752-153X-7-182-S1.zip › GeneratedImages/dzc_output_images/gjtccjesi160113_files/10/0_1.jpg]

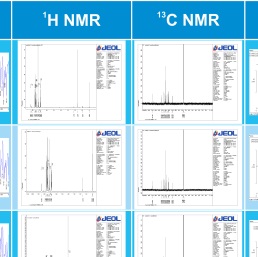

Supplement: Additional file 1 — DeepZoom: 4-methylidene oxindole synthesis and characterization. Microsoft Silverlight plug-in is required to view this. Use + and - buttons or scroll wheel to zoom in and out of image to view individual spectra. Use home button to reset view to full image. Click table cells to follow link to relevant section of electronic lab notebook for full supporting data (spectral assignments etc). [file 1752-153X-7-182-S1.zip › GeneratedImages/dzc_output_images/gjtccjesi160113_files/10/1_0.jpg]

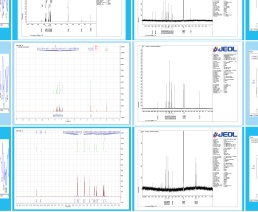

Supplement: Additional file 1 — DeepZoom: 4-methylidene oxindole synthesis and characterization. Microsoft Silverlight plug-in is required to view this. Use + and - buttons or scroll wheel to zoom in and out of image to view individual spectra. Use home button to reset view to full image. Click table cells to follow link to relevant section of electronic lab notebook for full supporting data (spectral assignments etc). [file 1752-153X-7-182-S1.zip › GeneratedImages/dzc_output_images/gjtccjesi160113_files/10/1_1.jpg]

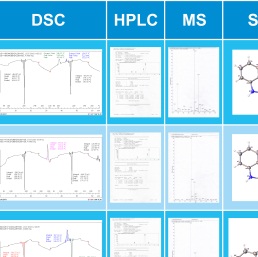

Supplement: Additional file 1 — DeepZoom: 4-methylidene oxindole synthesis and characterization. Microsoft Silverlight plug-in is required to view this. Use + and - buttons or scroll wheel to zoom in and out of image to view individual spectra. Use home button to reset view to full image. Click table cells to follow link to relevant section of electronic lab notebook for full supporting data (spectral assignments etc). [file 1752-153X-7-182-S1.zip › GeneratedImages/dzc_output_images/gjtccjesi160113_files/10/2_0.jpg]

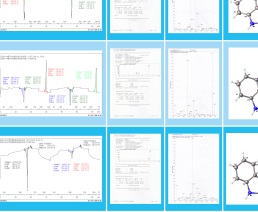

Supplement: Additional file 1 — DeepZoom: 4-methylidene oxindole synthesis and characterization. Microsoft Silverlight plug-in is required to view this. Use + and - buttons or scroll wheel to zoom in and out of image to view individual spectra. Use home button to reset view to full image. Click table cells to follow link to relevant section of electronic lab notebook for full supporting data (spectral assignments etc). [file 1752-153X-7-182-S1.zip › GeneratedImages/dzc_output_images/gjtccjesi160113_files/10/2_1.jpg]

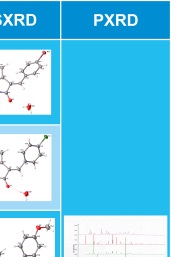

Supplement: Additional file 1 — DeepZoom: 4-methylidene oxindole synthesis and characterization. Microsoft Silverlight plug-in is required to view this. Use + and - buttons or scroll wheel to zoom in and out of image to view individual spectra. Use home button to reset view to full image. Click table cells to follow link to relevant section of electronic lab notebook for full supporting data (spectral assignments etc). [file 1752-153X-7-182-S1.zip › GeneratedImages/dzc_output_images/gjtccjesi160113_files/10/3_0.jpg]

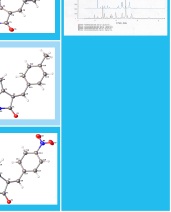

Supplement: Additional file 1 — DeepZoom: 4-methylidene oxindole synthesis and characterization. Microsoft Silverlight plug-in is required to view this. Use + and - buttons or scroll wheel to zoom in and out of image to view individual spectra. Use home button to reset view to full image. Click table cells to follow link to relevant section of electronic lab notebook for full supporting data (spectral assignments etc). [file 1752-153X-7-182-S1.zip › GeneratedImages/dzc_output_images/gjtccjesi160113_files/10/3_1.jpg]

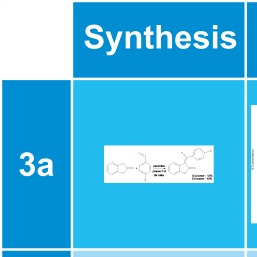

Supplement: Additional file 1 — DeepZoom: 4-methylidene oxindole synthesis and characterization. Microsoft Silverlight plug-in is required to view this. Use + and - buttons or scroll wheel to zoom in and out of image to view individual spectra. Use home button to reset view to full image. Click table cells to follow link to relevant section of electronic lab notebook for full supporting data (spectral assignments etc). [file 1752-153X-7-182-S1.zip › GeneratedImages/dzc_output_images/gjtccjesi160113_files/11/0_0.jpg]

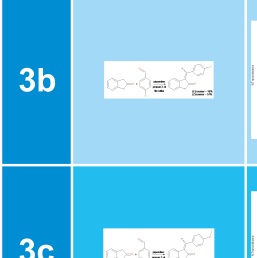

Supplement: Additional file 1 — DeepZoom: 4-methylidene oxindole synthesis and characterization. Microsoft Silverlight plug-in is required to view this. Use + and - buttons or scroll wheel to zoom in and out of image to view individual spectra. Use home button to reset view to full image. Click table cells to follow link to relevant section of electronic lab notebook for full supporting data (spectral assignments etc). [file 1752-153X-7-182-S1.zip › GeneratedImages/dzc_output_images/gjtccjesi160113_files/11/0_1.jpg]

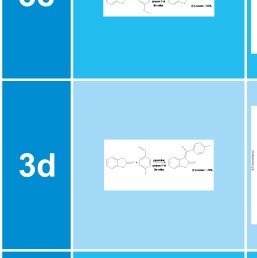

Supplement: Additional file 1 — DeepZoom: 4-methylidene oxindole synthesis and characterization. Microsoft Silverlight plug-in is required to view this. Use + and - buttons or scroll wheel to zoom in and out of image to view individual spectra. Use home button to reset view to full image. Click table cells to follow link to relevant section of electronic lab notebook for full supporting data (spectral assignments etc). [file 1752-153X-7-182-S1.zip › GeneratedImages/dzc_output_images/gjtccjesi160113_files/11/0_2.jpg]

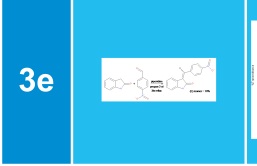

Supplement: Additional file 1 — DeepZoom: 4-methylidene oxindole synthesis and characterization. Microsoft Silverlight plug-in is required to view this. Use + and - buttons or scroll wheel to zoom in and out of image to view individual spectra. Use home button to reset view to full image. Click table cells to follow link to relevant section of electronic lab notebook for full supporting data (spectral assignments etc). [file 1752-153X-7-182-S1.zip › GeneratedImages/dzc_output_images/gjtccjesi160113_files/11/0_3.jpg]

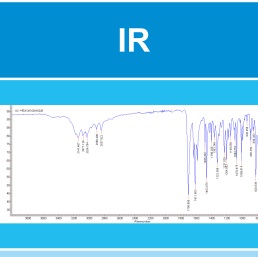

Supplement: Additional file 1 — DeepZoom: 4-methylidene oxindole synthesis and characterization. Microsoft Silverlight plug-in is required to view this. Use + and - buttons or scroll wheel to zoom in and out of image to view individual spectra. Use home button to reset view to full image. Click table cells to follow link to relevant section of electronic lab notebook for full supporting data (spectral assignments etc). [file 1752-153X-7-182-S1.zip › GeneratedImages/dzc_output_images/gjtccjesi160113_files/11/1_0.jpg]

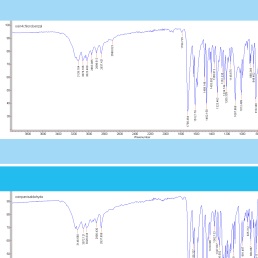

Supplement: Additional file 1 — DeepZoom: 4-methylidene oxindole synthesis and characterization. Microsoft Silverlight plug-in is required to view this. Use + and - buttons or scroll wheel to zoom in and out of image to view individual spectra. Use home button to reset view to full image. Click table cells to follow link to relevant section of electronic lab notebook for full supporting data (spectral assignments etc). [file 1752-153X-7-182-S1.zip › GeneratedImages/dzc_output_images/gjtccjesi160113_files/11/1_1.jpg]

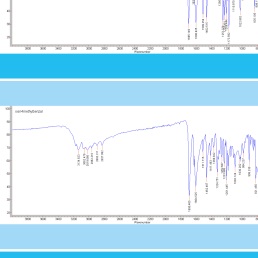

Supplement: Additional file 1 — DeepZoom: 4-methylidene oxindole synthesis and characterization. Microsoft Silverlight plug-in is required to view this. Use + and - buttons or scroll wheel to zoom in and out of image to view individual spectra. Use home button to reset view to full image. Click table cells to follow link to relevant section of electronic lab notebook for full supporting data (spectral assignments etc). [file 1752-153X-7-182-S1.zip › GeneratedImages/dzc_output_images/gjtccjesi160113_files/11/1_2.jpg]

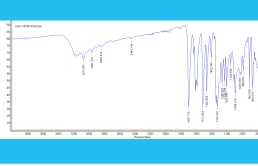

Supplement: Additional file 1 — DeepZoom: 4-methylidene oxindole synthesis and characterization. Microsoft Silverlight plug-in is required to view this. Use + and - buttons or scroll wheel to zoom in and out of image to view individual spectra. Use home button to reset view to full image. Click table cells to follow link to relevant section of electronic lab notebook for full supporting data (spectral assignments etc). [file 1752-153X-7-182-S1.zip › GeneratedImages/dzc_output_images/gjtccjesi160113_files/11/1_3.jpg]

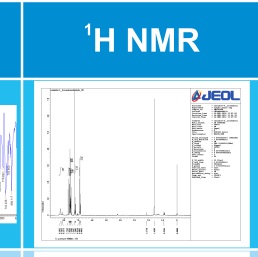

Supplement: Additional file 1 — DeepZoom: 4-methylidene oxindole synthesis and characterization. Microsoft Silverlight plug-in is required to view this. Use + and - buttons or scroll wheel to zoom in and out of image to view individual spectra. Use home button to reset view to full image. Click table cells to follow link to relevant section of electronic lab notebook for full supporting data (spectral assignments etc). [file 1752-153X-7-182-S1.zip › GeneratedImages/dzc_output_images/gjtccjesi160113_files/11/2_0.jpg]

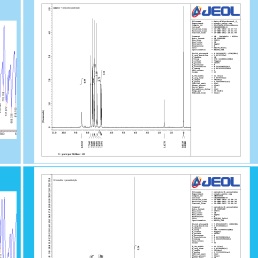

Supplement: Additional file 1 — DeepZoom: 4-methylidene oxindole synthesis and characterization. Microsoft Silverlight plug-in is required to view this. Use + and - buttons or scroll wheel to zoom in and out of image to view individual spectra. Use home button to reset view to full image. Click table cells to follow link to relevant section of electronic lab notebook for full supporting data (spectral assignments etc). [file 1752-153X-7-182-S1.zip › GeneratedImages/dzc_output_images/gjtccjesi160113_files/11/2_1.jpg]

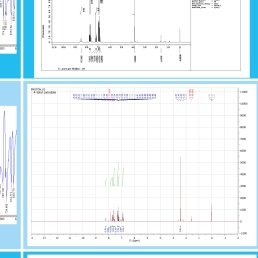

Supplement: Additional file 1 — DeepZoom: 4-methylidene oxindole synthesis and characterization. Microsoft Silverlight plug-in is required to view this. Use + and - buttons or scroll wheel to zoom in and out of image to view individual spectra. Use home button to reset view to full image. Click table cells to follow link to relevant section of electronic lab notebook for full supporting data (spectral assignments etc). [file 1752-153X-7-182-S1.zip › GeneratedImages/dzc_output_images/gjtccjesi160113_files/11/2_2.jpg]

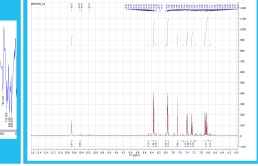

Supplement: Additional file 1 — DeepZoom: 4-methylidene oxindole synthesis and characterization. Microsoft Silverlight plug-in is required to view this. Use + and - buttons or scroll wheel to zoom in and out of image to view individual spectra. Use home button to reset view to full image. Click table cells to follow link to relevant section of electronic lab notebook for full supporting data (spectral assignments etc). [file 1752-153X-7-182-S1.zip › GeneratedImages/dzc_output_images/gjtccjesi160113_files/11/2_3.jpg]

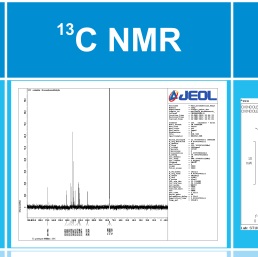

Supplement: Additional file 1 — DeepZoom: 4-methylidene oxindole synthesis and characterization. Microsoft Silverlight plug-in is required to view this. Use + and - buttons or scroll wheel to zoom in and out of image to view individual spectra. Use home button to reset view to full image. Click table cells to follow link to relevant section of electronic lab notebook for full supporting data (spectral assignments etc). [file 1752-153X-7-182-S1.zip › GeneratedImages/dzc_output_images/gjtccjesi160113_files/11/3_0.jpg]

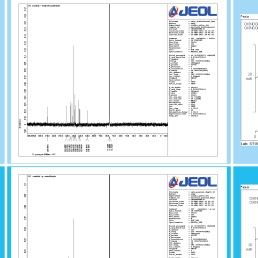

Supplement: Additional file 1 — DeepZoom: 4-methylidene oxindole synthesis and characterization. Microsoft Silverlight plug-in is required to view this. Use + and - buttons or scroll wheel to zoom in and out of image to view individual spectra. Use home button to reset view to full image. Click table cells to follow link to relevant section of electronic lab notebook for full supporting data (spectral assignments etc). [file 1752-153X-7-182-S1.zip › GeneratedImages/dzc_output_images/gjtccjesi160113_files/11/3_1.jpg]

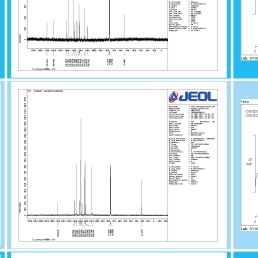

Supplement: Additional file 1 — DeepZoom: 4-methylidene oxindole synthesis and characterization. Microsoft Silverlight plug-in is required to view this. Use + and - buttons or scroll wheel to zoom in and out of image to view individual spectra. Use home button to reset view to full image. Click table cells to follow link to relevant section of electronic lab notebook for full supporting data (spectral assignments etc). [file 1752-153X-7-182-S1.zip › GeneratedImages/dzc_output_images/gjtccjesi160113_files/11/3_2.jpg]

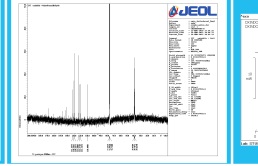

Supplement: Additional file 1 — DeepZoom: 4-methylidene oxindole synthesis and characterization. Microsoft Silverlight plug-in is required to view this. Use + and - buttons or scroll wheel to zoom in and out of image to view individual spectra. Use home button to reset view to full image. Click table cells to follow link to relevant section of electronic lab notebook for full supporting data (spectral assignments etc). [file 1752-153X-7-182-S1.zip › GeneratedImages/dzc_output_images/gjtccjesi160113_files/11/3_3.jpg]

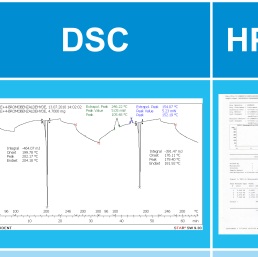

Supplement: Additional file 1 — DeepZoom: 4-methylidene oxindole synthesis and characterization. Microsoft Silverlight plug-in is required to view this. Use + and - buttons or scroll wheel to zoom in and out of image to view individual spectra. Use home button to reset view to full image. Click table cells to follow link to relevant section of electronic lab notebook for full supporting data (spectral assignments etc). [file 1752-153X-7-182-S1.zip › GeneratedImages/dzc_output_images/gjtccjesi160113_files/11/4_0.jpg]

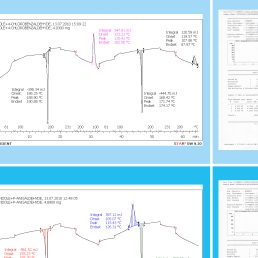

Supplement: Additional file 1 — DeepZoom: 4-methylidene oxindole synthesis and characterization. Microsoft Silverlight plug-in is required to view this. Use + and - buttons or scroll wheel to zoom in and out of image to view individual spectra. Use home button to reset view to full image. Click table cells to follow link to relevant section of electronic lab notebook for full supporting data (spectral assignments etc). [file 1752-153X-7-182-S1.zip › GeneratedImages/dzc_output_images/gjtccjesi160113_files/11/4_1.jpg]

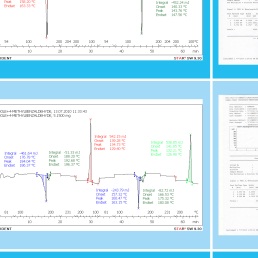

Supplement: Additional file 1 — DeepZoom: 4-methylidene oxindole synthesis and characterization. Microsoft Silverlight plug-in is required to view this. Use + and - buttons or scroll wheel to zoom in and out of image to view individual spectra. Use home button to reset view to full image. Click table cells to follow link to relevant section of electronic lab notebook for full supporting data (spectral assignments etc). [file 1752-153X-7-182-S1.zip › GeneratedImages/dzc_output_images/gjtccjesi160113_files/11/4_2.jpg]

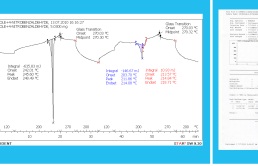

Supplement: Additional file 1 — DeepZoom: 4-methylidene oxindole synthesis and characterization. Microsoft Silverlight plug-in is required to view this. Use + and - buttons or scroll wheel to zoom in and out of image to view individual spectra. Use home button to reset view to full image. Click table cells to follow link to relevant section of electronic lab notebook for full supporting data (spectral assignments etc). [file 1752-153X-7-182-S1.zip › GeneratedImages/dzc_output_images/gjtccjesi160113_files/11/4_3.jpg]

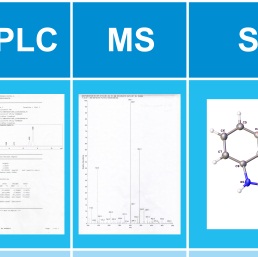

Supplement: Additional file 1 — DeepZoom: 4-methylidene oxindole synthesis and characterization. Microsoft Silverlight plug-in is required to view this. Use + and - buttons or scroll wheel to zoom in and out of image to view individual spectra. Use home button to reset view to full image. Click table cells to follow link to relevant section of electronic lab notebook for full supporting data (spectral assignments etc). [file 1752-153X-7-182-S1.zip › GeneratedImages/dzc_output_images/gjtccjesi160113_files/11/5_0.jpg]

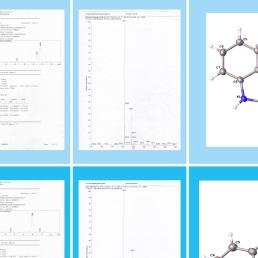

Supplement: Additional file 1 — DeepZoom: 4-methylidene oxindole synthesis and characterization. Microsoft Silverlight plug-in is required to view this. Use + and - buttons or scroll wheel to zoom in and out of image to view individual spectra. Use home button to reset view to full image. Click table cells to follow link to relevant section of electronic lab notebook for full supporting data (spectral assignments etc). [file 1752-153X-7-182-S1.zip › GeneratedImages/dzc_output_images/gjtccjesi160113_files/11/5_1.jpg]

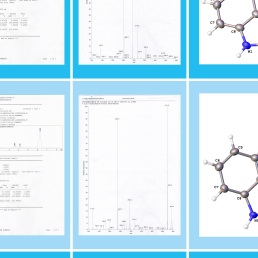

Supplement: Additional file 1 — DeepZoom: 4-methylidene oxindole synthesis and characterization. Microsoft Silverlight plug-in is required to view this. Use + and - buttons or scroll wheel to zoom in and out of image to view individual spectra. Use home button to reset view to full image. Click table cells to follow link to relevant section of electronic lab notebook for full supporting data (spectral assignments etc). [file 1752-153X-7-182-S1.zip › GeneratedImages/dzc_output_images/gjtccjesi160113_files/11/5_2.jpg]

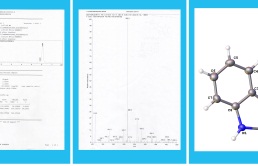

Supplement: Additional file 1 — DeepZoom: 4-methylidene oxindole synthesis and characterization. Microsoft Silverlight plug-in is required to view this. Use + and - buttons or scroll wheel to zoom in and out of image to view individual spectra. Use home button to reset view to full image. Click table cells to follow link to relevant section of electronic lab notebook for full supporting data (spectral assignments etc). [file 1752-153X-7-182-S1.zip › GeneratedImages/dzc_output_images/gjtccjesi160113_files/11/5_3.jpg]

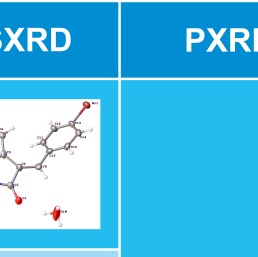

Supplement: Additional file 1 — DeepZoom: 4-methylidene oxindole synthesis and characterization. Microsoft Silverlight plug-in is required to view this. Use + and - buttons or scroll wheel to zoom in and out of image to view individual spectra. Use home button to reset view to full image. Click table cells to follow link to relevant section of electronic lab notebook for full supporting data (spectral assignments etc). [file 1752-153X-7-182-S1.zip › GeneratedImages/dzc_output_images/gjtccjesi160113_files/11/6_0.jpg]

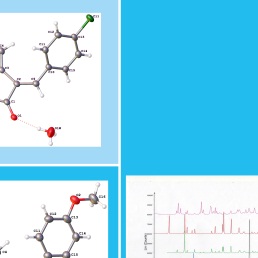

Supplement: Additional file 1 — DeepZoom: 4-methylidene oxindole synthesis and characterization. Microsoft Silverlight plug-in is required to view this. Use + and - buttons or scroll wheel to zoom in and out of image to view individual spectra. Use home button to reset view to full image. Click table cells to follow link to relevant section of electronic lab notebook for full supporting data (spectral assignments etc). [file 1752-153X-7-182-S1.zip › GeneratedImages/dzc_output_images/gjtccjesi160113_files/11/6_1.jpg]

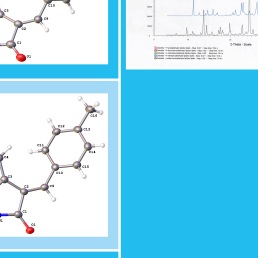

Supplement: Additional file 1 — DeepZoom: 4-methylidene oxindole synthesis and characterization. Microsoft Silverlight plug-in is required to view this. Use + and - buttons or scroll wheel to zoom in and out of image to view individual spectra. Use home button to reset view to full image. Click table cells to follow link to relevant section of electronic lab notebook for full supporting data (spectral assignments etc). [file 1752-153X-7-182-S1.zip › GeneratedImages/dzc_output_images/gjtccjesi160113_files/11/6_2.jpg]

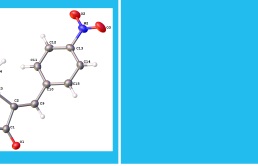

Supplement: Additional file 1 — DeepZoom: 4-methylidene oxindole synthesis and characterization. Microsoft Silverlight plug-in is required to view this. Use + and - buttons or scroll wheel to zoom in and out of image to view individual spectra. Use home button to reset view to full image. Click table cells to follow link to relevant section of electronic lab notebook for full supporting data (spectral assignments etc). [file 1752-153X-7-182-S1.zip › GeneratedImages/dzc_output_images/gjtccjesi160113_files/11/6_3.jpg]

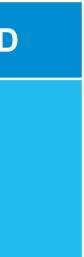

Supplement: Additional file 1 — DeepZoom: 4-methylidene oxindole synthesis and characterization. Microsoft Silverlight plug-in is required to view this. Use + and - buttons or scroll wheel to zoom in and out of image to view individual spectra. Use home button to reset view to full image. Click table cells to follow link to relevant section of electronic lab notebook for full supporting data (spectral assignments etc). [file 1752-153X-7-182-S1.zip › GeneratedImages/dzc_output_images/gjtccjesi160113_files/11/7_0.jpg]

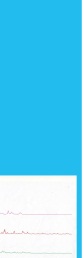

Supplement: Additional file 1 — DeepZoom: 4-methylidene oxindole synthesis and characterization. Microsoft Silverlight plug-in is required to view this. Use + and - buttons or scroll wheel to zoom in and out of image to view individual spectra. Use home button to reset view to full image. Click table cells to follow link to relevant section of electronic lab notebook for full supporting data (spectral assignments etc). [file 1752-153X-7-182-S1.zip › GeneratedImages/dzc_output_images/gjtccjesi160113_files/11/7_1.jpg]

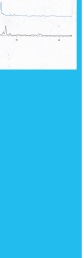

Supplement: Additional file 1 — DeepZoom: 4-methylidene oxindole synthesis and characterization. Microsoft Silverlight plug-in is required to view this. Use + and - buttons or scroll wheel to zoom in and out of image to view individual spectra. Use home button to reset view to full image. Click table cells to follow link to relevant section of electronic lab notebook for full supporting data (spectral assignments etc). [file 1752-153X-7-182-S1.zip › GeneratedImages/dzc_output_images/gjtccjesi160113_files/11/7_2.jpg]

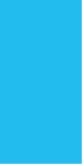

Supplement: Additional file 1 — DeepZoom: 4-methylidene oxindole synthesis and characterization. Microsoft Silverlight plug-in is required to view this. Use + and - buttons or scroll wheel to zoom in and out of image to view individual spectra. Use home button to reset view to full image. Click table cells to follow link to relevant section of electronic lab notebook for full supporting data (spectral assignments etc). [file 1752-153X-7-182-S1.zip › GeneratedImages/dzc_output_images/gjtccjesi160113_files/11/7_3.jpg]

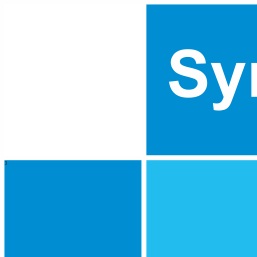

Supplement: Additional file 1 — DeepZoom: 4-methylidene oxindole synthesis and characterization. Microsoft Silverlight plug-in is required to view this. Use + and - buttons or scroll wheel to zoom in and out of image to view individual spectra. Use home button to reset view to full image. Click table cells to follow link to relevant section of electronic lab notebook for full supporting data (spectral assignments etc). [file 1752-153X-7-182-S1.zip › GeneratedImages/dzc_output_images/gjtccjesi160113_files/12/0_0.jpg]

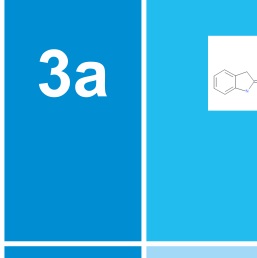

Supplement: Additional file 1 — DeepZoom: 4-methylidene oxindole synthesis and characterization. Microsoft Silverlight plug-in is required to view this. Use + and - buttons or scroll wheel to zoom in and out of image to view individual spectra. Use home button to reset view to full image. Click table cells to follow link to relevant section of electronic lab notebook for full supporting data (spectral assignments etc). [file 1752-153X-7-182-S1.zip › GeneratedImages/dzc_output_images/gjtccjesi160113_files/12/0_1.jpg]

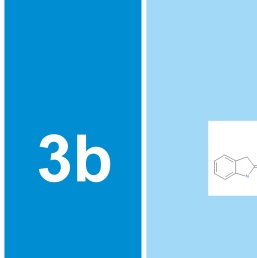

Supplement: Additional file 1 — DeepZoom: 4-methylidene oxindole synthesis and characterization. Microsoft Silverlight plug-in is required to view this. Use + and - buttons or scroll wheel to zoom in and out of image to view individual spectra. Use home button to reset view to full image. Click table cells to follow link to relevant section of electronic lab notebook for full supporting data (spectral assignments etc). [file 1752-153X-7-182-S1.zip › GeneratedImages/dzc_output_images/gjtccjesi160113_files/12/0_2.jpg]

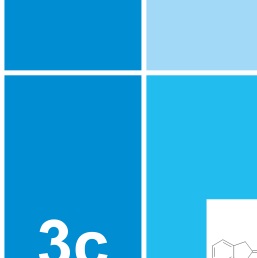

Supplement: Additional file 1 — DeepZoom: 4-methylidene oxindole synthesis and characterization. Microsoft Silverlight plug-in is required to view this. Use + and - buttons or scroll wheel to zoom in and out of image to view individual spectra. Use home button to reset view to full image. Click table cells to follow link to relevant section of electronic lab notebook for full supporting data (spectral assignments etc). [file 1752-153X-7-182-S1.zip › GeneratedImages/dzc_output_images/gjtccjesi160113_files/12/0_3.jpg]

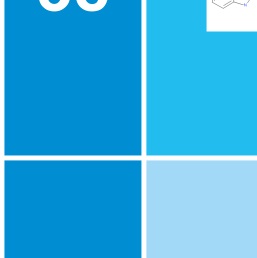

Supplement: Additional file 1 — DeepZoom: 4-methylidene oxindole synthesis and characterization. Microsoft Silverlight plug-in is required to view this. Use + and - buttons or scroll wheel to zoom in and out of image to view individual spectra. Use home button to reset view to full image. Click table cells to follow link to relevant section of electronic lab notebook for full supporting data (spectral assignments etc). [file 1752-153X-7-182-S1.zip › GeneratedImages/dzc_output_images/gjtccjesi160113_files/12/0_4.jpg]

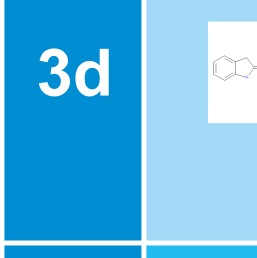

Supplement: Additional file 1 — DeepZoom: 4-methylidene oxindole synthesis and characterization. Microsoft Silverlight plug-in is required to view this. Use + and - buttons or scroll wheel to zoom in and out of image to view individual spectra. Use home button to reset view to full image. Click table cells to follow link to relevant section of electronic lab notebook for full supporting data (spectral assignments etc). [file 1752-153X-7-182-S1.zip › GeneratedImages/dzc_output_images/gjtccjesi160113_files/12/0_5.jpg]

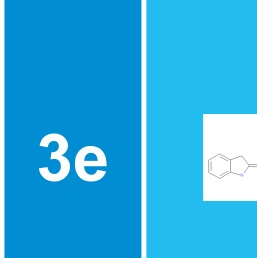

Supplement: Additional file 1 — DeepZoom: 4-methylidene oxindole synthesis and characterization. Microsoft Silverlight plug-in is required to view this. Use + and - buttons or scroll wheel to zoom in and out of image to view individual spectra. Use home button to reset view to full image. Click table cells to follow link to relevant section of electronic lab notebook for full supporting data (spectral assignments etc). [file 1752-153X-7-182-S1.zip › GeneratedImages/dzc_output_images/gjtccjesi160113_files/12/0_6.jpg]

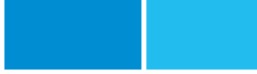

Supplement: Additional file 1 — DeepZoom: 4-methylidene oxindole synthesis and characterization. Microsoft Silverlight plug-in is required to view this. Use + and - buttons or scroll wheel to zoom in and out of image to view individual spectra. Use home button to reset view to full image. Click table cells to follow link to relevant section of electronic lab notebook for full supporting data (spectral assignments etc). [file 1752-153X-7-182-S1.zip › GeneratedImages/dzc_output_images/gjtccjesi160113_files/12/0_7.jpg]

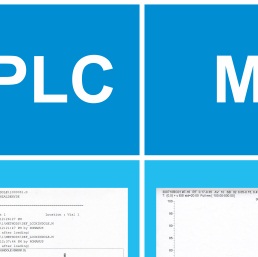

Supplement: Additional file 1 — DeepZoom: 4-methylidene oxindole synthesis and characterization. Microsoft Silverlight plug-in is required to view this. Use + and - buttons or scroll wheel to zoom in and out of image to view individual spectra. Use home button to reset view to full image. Click table cells to follow link to relevant section of electronic lab notebook for full supporting data (spectral assignments etc). [file 1752-153X-7-182-S1.zip › GeneratedImages/dzc_output_images/gjtccjesi160113_files/12/10_0.jpg]

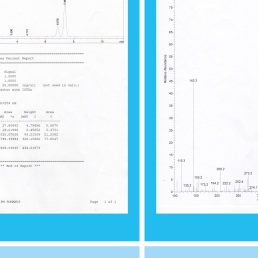

Supplement: Additional file 1 — DeepZoom: 4-methylidene oxindole synthesis and characterization. Microsoft Silverlight plug-in is required to view this. Use + and - buttons or scroll wheel to zoom in and out of image to view individual spectra. Use home button to reset view to full image. Click table cells to follow link to relevant section of electronic lab notebook for full supporting data (spectral assignments etc). [file 1752-153X-7-182-S1.zip › GeneratedImages/dzc_output_images/gjtccjesi160113_files/12/10_1.jpg]

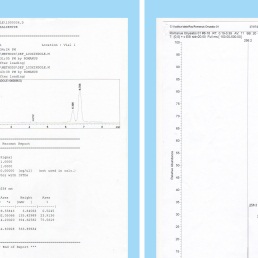

Supplement: Additional file 1 — DeepZoom: 4-methylidene oxindole synthesis and characterization. Microsoft Silverlight plug-in is required to view this. Use + and - buttons or scroll wheel to zoom in and out of image to view individual spectra. Use home button to reset view to full image. Click table cells to follow link to relevant section of electronic lab notebook for full supporting data (spectral assignments etc). [file 1752-153X-7-182-S1.zip › GeneratedImages/dzc_output_images/gjtccjesi160113_files/12/10_2.jpg]

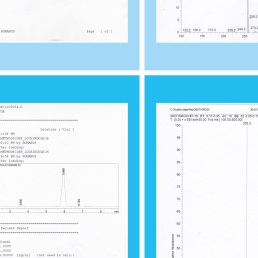

Supplement: Additional file 1 — DeepZoom: 4-methylidene oxindole synthesis and characterization. Microsoft Silverlight plug-in is required to view this. Use + and - buttons or scroll wheel to zoom in and out of image to view individual spectra. Use home button to reset view to full image. Click table cells to follow link to relevant section of electronic lab notebook for full supporting data (spectral assignments etc). [file 1752-153X-7-182-S1.zip › GeneratedImages/dzc_output_images/gjtccjesi160113_files/12/10_3.jpg]

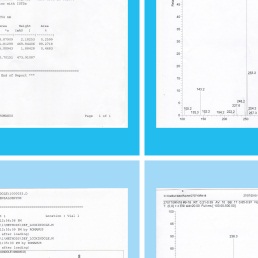

Supplement: Additional file 1 — DeepZoom: 4-methylidene oxindole synthesis and characterization. Microsoft Silverlight plug-in is required to view this. Use + and - buttons or scroll wheel to zoom in and out of image to view individual spectra. Use home button to reset view to full image. Click table cells to follow link to relevant section of electronic lab notebook for full supporting data (spectral assignments etc). [file 1752-153X-7-182-S1.zip › GeneratedImages/dzc_output_images/gjtccjesi160113_files/12/10_4.jpg]

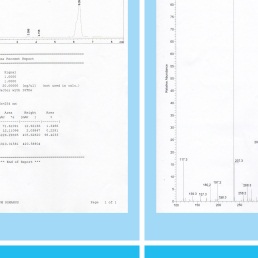

Supplement: Additional file 1 — DeepZoom: 4-methylidene oxindole synthesis and characterization. Microsoft Silverlight plug-in is required to view this. Use + and - buttons or scroll wheel to zoom in and out of image to view individual spectra. Use home button to reset view to full image. Click table cells to follow link to relevant section of electronic lab notebook for full supporting data (spectral assignments etc). [file 1752-153X-7-182-S1.zip › GeneratedImages/dzc_output_images/gjtccjesi160113_files/12/10_5.jpg]

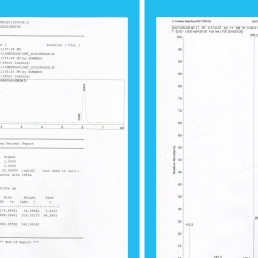

Supplement: Additional file 1 — DeepZoom: 4-methylidene oxindole synthesis and characterization. Microsoft Silverlight plug-in is required to view this. Use + and - buttons or scroll wheel to zoom in and out of image to view individual spectra. Use home button to reset view to full image. Click table cells to follow link to relevant section of electronic lab notebook for full supporting data (spectral assignments etc). [file 1752-153X-7-182-S1.zip › GeneratedImages/dzc_output_images/gjtccjesi160113_files/12/10_6.jpg]

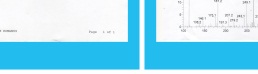

Supplement: Additional file 1 — DeepZoom: 4-methylidene oxindole synthesis and characterization. Microsoft Silverlight plug-in is required to view this. Use + and - buttons or scroll wheel to zoom in and out of image to view individual spectra. Use home button to reset view to full image. Click table cells to follow link to relevant section of electronic lab notebook for full supporting data (spectral assignments etc). [file 1752-153X-7-182-S1.zip › GeneratedImages/dzc_output_images/gjtccjesi160113_files/12/10_7.jpg]

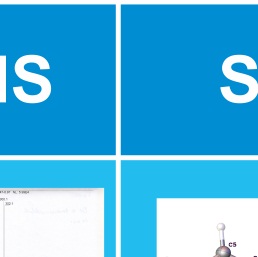

Supplement: Additional file 1 — DeepZoom: 4-methylidene oxindole synthesis and characterization. Microsoft Silverlight plug-in is required to view this. Use + and - buttons or scroll wheel to zoom in and out of image to view individual spectra. Use home button to reset view to full image. Click table cells to follow link to relevant section of electronic lab notebook for full supporting data (spectral assignments etc). [file 1752-153X-7-182-S1.zip › GeneratedImages/dzc_output_images/gjtccjesi160113_files/12/11_0.jpg]

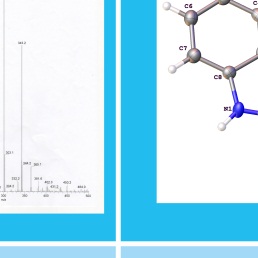

Supplement: Additional file 1 — DeepZoom: 4-methylidene oxindole synthesis and characterization. Microsoft Silverlight plug-in is required to view this. Use + and - buttons or scroll wheel to zoom in and out of image to view individual spectra. Use home button to reset view to full image. Click table cells to follow link to relevant section of electronic lab notebook for full supporting data (spectral assignments etc). [file 1752-153X-7-182-S1.zip › GeneratedImages/dzc_output_images/gjtccjesi160113_files/12/11_1.jpg]

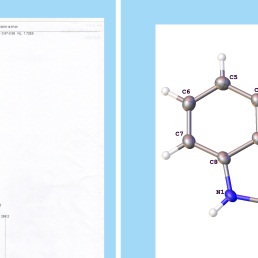

Supplement: Additional file 1 — DeepZoom: 4-methylidene oxindole synthesis and characterization. Microsoft Silverlight plug-in is required to view this. Use + and - buttons or scroll wheel to zoom in and out of image to view individual spectra. Use home button to reset view to full image. Click table cells to follow link to relevant section of electronic lab notebook for full supporting data (spectral assignments etc). [file 1752-153X-7-182-S1.zip › GeneratedImages/dzc_output_images/gjtccjesi160113_files/12/11_2.jpg]

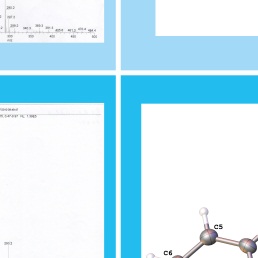

Supplement: Additional file 1 — DeepZoom: 4-methylidene oxindole synthesis and characterization. Microsoft Silverlight plug-in is required to view this. Use + and - buttons or scroll wheel to zoom in and out of image to view individual spectra. Use home button to reset view to full image. Click table cells to follow link to relevant section of electronic lab notebook for full supporting data (spectral assignments etc). [file 1752-153X-7-182-S1.zip › GeneratedImages/dzc_output_images/gjtccjesi160113_files/12/11_3.jpg]

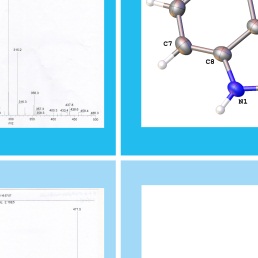

Supplement: Additional file 1 — DeepZoom: 4-methylidene oxindole synthesis and characterization. Microsoft Silverlight plug-in is required to view this. Use + and - buttons or scroll wheel to zoom in and out of image to view individual spectra. Use home button to reset view to full image. Click table cells to follow link to relevant section of electronic lab notebook for full supporting data (spectral assignments etc). [file 1752-153X-7-182-S1.zip › GeneratedImages/dzc_output_images/gjtccjesi160113_files/12/11_4.jpg]

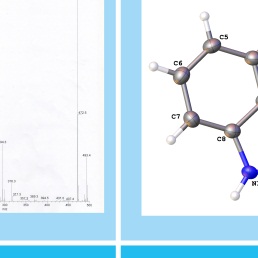

Supplement: Additional file 1 — DeepZoom: 4-methylidene oxindole synthesis and characterization. Microsoft Silverlight plug-in is required to view this. Use + and - buttons or scroll wheel to zoom in and out of image to view individual spectra. Use home button to reset view to full image. Click table cells to follow link to relevant section of electronic lab notebook for full supporting data (spectral assignments etc). [file 1752-153X-7-182-S1.zip › GeneratedImages/dzc_output_images/gjtccjesi160113_files/12/11_5.jpg]

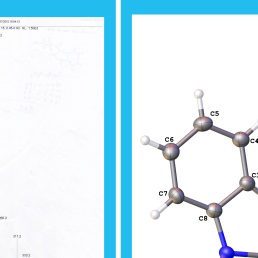

Supplement: Additional file 1 — DeepZoom: 4-methylidene oxindole synthesis and characterization. Microsoft Silverlight plug-in is required to view this. Use + and - buttons or scroll wheel to zoom in and out of image to view individual spectra. Use home button to reset view to full image. Click table cells to follow link to relevant section of electronic lab notebook for full supporting data (spectral assignments etc). [file 1752-153X-7-182-S1.zip › GeneratedImages/dzc_output_images/gjtccjesi160113_files/12/11_6.jpg]

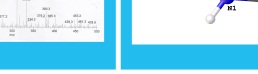

Supplement: Additional file 1 — DeepZoom: 4-methylidene oxindole synthesis and characterization. Microsoft Silverlight plug-in is required to view this. Use + and - buttons or scroll wheel to zoom in and out of image to view individual spectra. Use home button to reset view to full image. Click table cells to follow link to relevant section of electronic lab notebook for full supporting data (spectral assignments etc). [file 1752-153X-7-182-S1.zip › GeneratedImages/dzc_output_images/gjtccjesi160113_files/12/11_7.jpg]

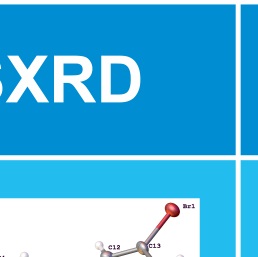

Supplement: Additional file 1 — DeepZoom: 4-methylidene oxindole synthesis and characterization. Microsoft Silverlight plug-in is required to view this. Use + and - buttons or scroll wheel to zoom in and out of image to view individual spectra. Use home button to reset view to full image. Click table cells to follow link to relevant section of electronic lab notebook for full supporting data (spectral assignments etc). [file 1752-153X-7-182-S1.zip › GeneratedImages/dzc_output_images/gjtccjesi160113_files/12/12_0.jpg]

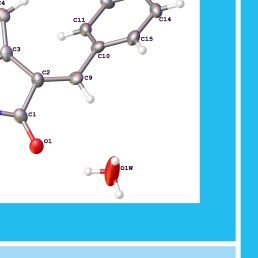

Supplement: Additional file 1 — DeepZoom: 4-methylidene oxindole synthesis and characterization. Microsoft Silverlight plug-in is required to view this. Use + and - buttons or scroll wheel to zoom in and out of image to view individual spectra. Use home button to reset view to full image. Click table cells to follow link to relevant section of electronic lab notebook for full supporting data (spectral assignments etc). [file 1752-153X-7-182-S1.zip › GeneratedImages/dzc_output_images/gjtccjesi160113_files/12/12_1.jpg]

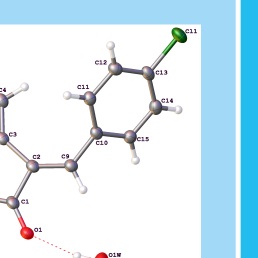

Supplement: Additional file 1 — DeepZoom: 4-methylidene oxindole synthesis and characterization. Microsoft Silverlight plug-in is required to view this. Use + and - buttons or scroll wheel to zoom in and out of image to view individual spectra. Use home button to reset view to full image. Click table cells to follow link to relevant section of electronic lab notebook for full supporting data (spectral assignments etc). [file 1752-153X-7-182-S1.zip › GeneratedImages/dzc_output_images/gjtccjesi160113_files/12/12_2.jpg]

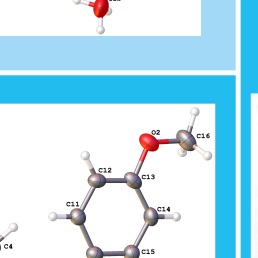

Supplement: Additional file 1 — DeepZoom: 4-methylidene oxindole synthesis and characterization. Microsoft Silverlight plug-in is required to view this. Use + and - buttons or scroll wheel to zoom in and out of image to view individual spectra. Use home button to reset view to full image. Click table cells to follow link to relevant section of electronic lab notebook for full supporting data (spectral assignments etc). [file 1752-153X-7-182-S1.zip › GeneratedImages/dzc_output_images/gjtccjesi160113_files/12/12_3.jpg]

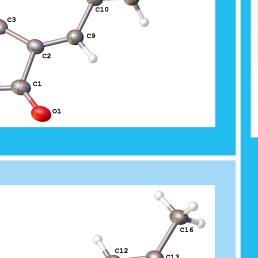

Supplement: Additional file 1 — DeepZoom: 4-methylidene oxindole synthesis and characterization. Microsoft Silverlight plug-in is required to view this. Use + and - buttons or scroll wheel to zoom in and out of image to view individual spectra. Use home button to reset view to full image. Click table cells to follow link to relevant section of electronic lab notebook for full supporting data (spectral assignments etc). [file 1752-153X-7-182-S1.zip › GeneratedImages/dzc_output_images/gjtccjesi160113_files/12/12_4.jpg]

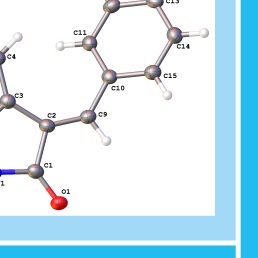

Supplement: Additional file 1 — DeepZoom: 4-methylidene oxindole synthesis and characterization. Microsoft Silverlight plug-in is required to view this. Use + and - buttons or scroll wheel to zoom in and out of image to view individual spectra. Use home button to reset view to full image. Click table cells to follow link to relevant section of electronic lab notebook for full supporting data (spectral assignments etc). [file 1752-153X-7-182-S1.zip › GeneratedImages/dzc_output_images/gjtccjesi160113_files/12/12_5.jpg]

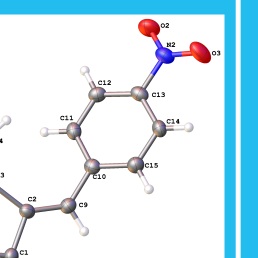

Supplement: Additional file 1 — DeepZoom: 4-methylidene oxindole synthesis and characterization. Microsoft Silverlight plug-in is required to view this. Use + and - buttons or scroll wheel to zoom in and out of image to view individual spectra. Use home button to reset view to full image. Click table cells to follow link to relevant section of electronic lab notebook for full supporting data (spectral assignments etc). [file 1752-153X-7-182-S1.zip › GeneratedImages/dzc_output_images/gjtccjesi160113_files/12/12_6.jpg]

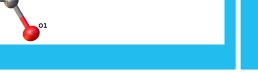

Supplement: Additional file 1 — DeepZoom: 4-methylidene oxindole synthesis and characterization. Microsoft Silverlight plug-in is required to view this. Use + and - buttons or scroll wheel to zoom in and out of image to view individual spectra. Use home button to reset view to full image. Click table cells to follow link to relevant section of electronic lab notebook for full supporting data (spectral assignments etc). [file 1752-153X-7-182-S1.zip › GeneratedImages/dzc_output_images/gjtccjesi160113_files/12/12_7.jpg]

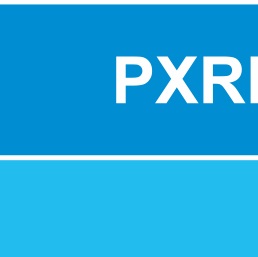

Supplement: Additional file 1 — DeepZoom: 4-methylidene oxindole synthesis and characterization. Microsoft Silverlight plug-in is required to view this. Use + and - buttons or scroll wheel to zoom in and out of image to view individual spectra. Use home button to reset view to full image. Click table cells to follow link to relevant section of electronic lab notebook for full supporting data (spectral assignments etc). [file 1752-153X-7-182-S1.zip › GeneratedImages/dzc_output_images/gjtccjesi160113_files/12/13_0.jpg]

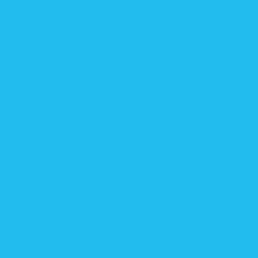

Supplement: Additional file 1 — DeepZoom: 4-methylidene oxindole synthesis and characterization. Microsoft Silverlight plug-in is required to view this. Use + and - buttons or scroll wheel to zoom in and out of image to view individual spectra. Use home button to reset view to full image. Click table cells to follow link to relevant section of electronic lab notebook for full supporting data (spectral assignments etc). [file 1752-153X-7-182-S1.zip › GeneratedImages/dzc_output_images/gjtccjesi160113_files/12/13_1.jpg]

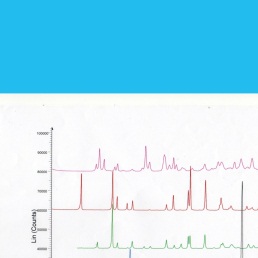

Supplement: Additional file 1 — DeepZoom: 4-methylidene oxindole synthesis and characterization. Microsoft Silverlight plug-in is required to view this. Use + and - buttons or scroll wheel to zoom in and out of image to view individual spectra. Use home button to reset view to full image. Click table cells to follow link to relevant section of electronic lab notebook for full supporting data (spectral assignments etc). [file 1752-153X-7-182-S1.zip › GeneratedImages/dzc_output_images/gjtccjesi160113_files/12/13_3.jpg]

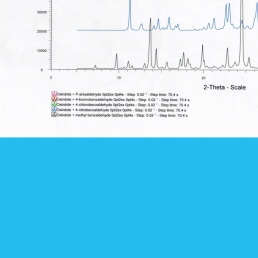

Supplement: Additional file 1 — DeepZoom: 4-methylidene oxindole synthesis and characterization. Microsoft Silverlight plug-in is required to view this. Use + and - buttons or scroll wheel to zoom in and out of image to view individual spectra. Use home button to reset view to full image. Click table cells to follow link to relevant section of electronic lab notebook for full supporting data (spectral assignments etc). [file 1752-153X-7-182-S1.zip › GeneratedImages/dzc_output_images/gjtccjesi160113_files/12/13_4.jpg]

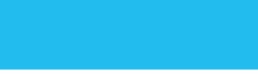

Supplement: Additional file 1 — DeepZoom: 4-methylidene oxindole synthesis and characterization. Microsoft Silverlight plug-in is required to view this. Use + and - buttons or scroll wheel to zoom in and out of image to view individual spectra. Use home button to reset view to full image. Click table cells to follow link to relevant section of electronic lab notebook for full supporting data (spectral assignments etc). [file 1752-153X-7-182-S1.zip › GeneratedImages/dzc_output_images/gjtccjesi160113_files/12/13_7.jpg]

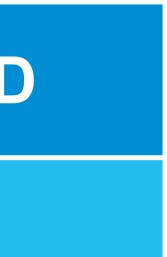

Supplement: Additional file 1 — DeepZoom: 4-methylidene oxindole synthesis and characterization. Microsoft Silverlight plug-in is required to view this. Use + and - buttons or scroll wheel to zoom in and out of image to view individual spectra. Use home button to reset view to full image. Click table cells to follow link to relevant section of electronic lab notebook for full supporting data (spectral assignments etc). [file 1752-153X-7-182-S1.zip › GeneratedImages/dzc_output_images/gjtccjesi160113_files/12/14_0.jpg]

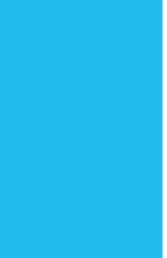

Supplement: Additional file 1 — DeepZoom: 4-methylidene oxindole synthesis and characterization. Microsoft Silverlight plug-in is required to view this. Use + and - buttons or scroll wheel to zoom in and out of image to view individual spectra. Use home button to reset view to full image. Click table cells to follow link to relevant section of electronic lab notebook for full supporting data (spectral assignments etc). [file 1752-153X-7-182-S1.zip › GeneratedImages/dzc_output_images/gjtccjesi160113_files/12/14_1.jpg]

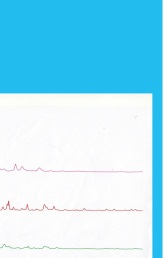

Supplement: Additional file 1 — DeepZoom: 4-methylidene oxindole synthesis and characterization. Microsoft Silverlight plug-in is required to view this. Use + and - buttons or scroll wheel to zoom in and out of image to view individual spectra. Use home button to reset view to full image. Click table cells to follow link to relevant section of electronic lab notebook for full supporting data (spectral assignments etc). [file 1752-153X-7-182-S1.zip › GeneratedImages/dzc_output_images/gjtccjesi160113_files/12/14_3.jpg]

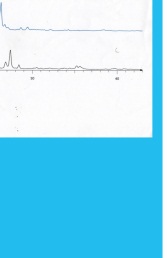

Supplement: Additional file 1 — DeepZoom: 4-methylidene oxindole synthesis and characterization. Microsoft Silverlight plug-in is required to view this. Use + and - buttons or scroll wheel to zoom in and out of image to view individual spectra. Use home button to reset view to full image. Click table cells to follow link to relevant section of electronic lab notebook for full supporting data (spectral assignments etc). [file 1752-153X-7-182-S1.zip › GeneratedImages/dzc_output_images/gjtccjesi160113_files/12/14_4.jpg]

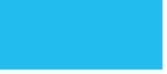

Supplement: Additional file 1 — DeepZoom: 4-methylidene oxindole synthesis and characterization. Microsoft Silverlight plug-in is required to view this. Use + and - buttons or scroll wheel to zoom in and out of image to view individual spectra. Use home button to reset view to full image. Click table cells to follow link to relevant section of electronic lab notebook for full supporting data (spectral assignments etc). [file 1752-153X-7-182-S1.zip › GeneratedImages/dzc_output_images/gjtccjesi160113_files/12/14_7.jpg]

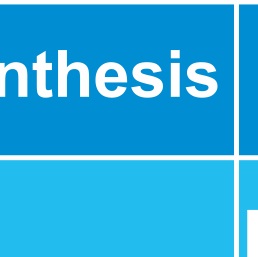

Supplement: Additional file 1 — DeepZoom: 4-methylidene oxindole synthesis and characterization. Microsoft Silverlight plug-in is required to view this. Use + and - buttons or scroll wheel to zoom in and out of image to view individual spectra. Use home button to reset view to full image. Click table cells to follow link to relevant section of electronic lab notebook for full supporting data (spectral assignments etc). [file 1752-153X-7-182-S1.zip › GeneratedImages/dzc_output_images/gjtccjesi160113_files/12/1_0.jpg]

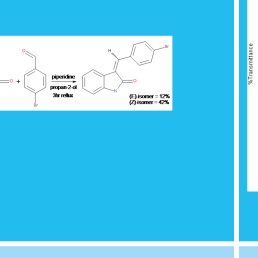

Supplement: Additional file 1 — DeepZoom: 4-methylidene oxindole synthesis and characterization. Microsoft Silverlight plug-in is required to view this. Use + and - buttons or scroll wheel to zoom in and out of image to view individual spectra. Use home button to reset view to full image. Click table cells to follow link to relevant section of electronic lab notebook for full supporting data (spectral assignments etc). [file 1752-153X-7-182-S1.zip › GeneratedImages/dzc_output_images/gjtccjesi160113_files/12/1_1.jpg]

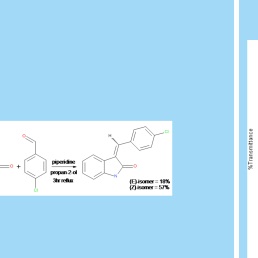

Supplement: Additional file 1 — DeepZoom: 4-methylidene oxindole synthesis and characterization. Microsoft Silverlight plug-in is required to view this. Use + and - buttons or scroll wheel to zoom in and out of image to view individual spectra. Use home button to reset view to full image. Click table cells to follow link to relevant section of electronic lab notebook for full supporting data (spectral assignments etc). [file 1752-153X-7-182-S1.zip › GeneratedImages/dzc_output_images/gjtccjesi160113_files/12/1_2.jpg]

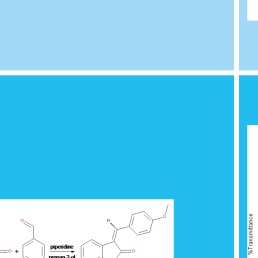

Supplement: Additional file 1 — DeepZoom: 4-methylidene oxindole synthesis and characterization. Microsoft Silverlight plug-in is required to view this. Use + and - buttons or scroll wheel to zoom in and out of image to view individual spectra. Use home button to reset view to full image. Click table cells to follow link to relevant section of electronic lab notebook for full supporting data (spectral assignments etc). [file 1752-153X-7-182-S1.zip › GeneratedImages/dzc_output_images/gjtccjesi160113_files/12/1_3.jpg]

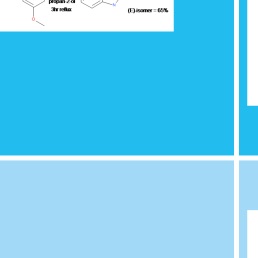

Supplement: Additional file 1 — DeepZoom: 4-methylidene oxindole synthesis and characterization. Microsoft Silverlight plug-in is required to view this. Use + and - buttons or scroll wheel to zoom in and out of image to view individual spectra. Use home button to reset view to full image. Click table cells to follow link to relevant section of electronic lab notebook for full supporting data (spectral assignments etc). [file 1752-153X-7-182-S1.zip › GeneratedImages/dzc_output_images/gjtccjesi160113_files/12/1_4.jpg]

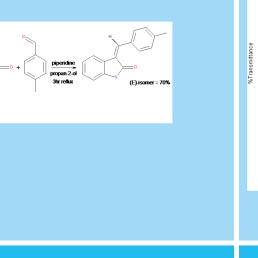

Supplement: Additional file 1 — DeepZoom: 4-methylidene oxindole synthesis and characterization. Microsoft Silverlight plug-in is required to view this. Use + and - buttons or scroll wheel to zoom in and out of image to view individual spectra. Use home button to reset view to full image. Click table cells to follow link to relevant section of electronic lab notebook for full supporting data (spectral assignments etc). [file 1752-153X-7-182-S1.zip › GeneratedImages/dzc_output_images/gjtccjesi160113_files/12/1_5.jpg]

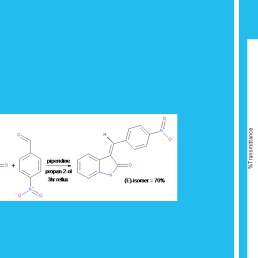

Supplement: Additional file 1 — DeepZoom: 4-methylidene oxindole synthesis and characterization. Microsoft Silverlight plug-in is required to view this. Use + and - buttons or scroll wheel to zoom in and out of image to view individual spectra. Use home button to reset view to full image. Click table cells to follow link to relevant section of electronic lab notebook for full supporting data (spectral assignments etc). [file 1752-153X-7-182-S1.zip › GeneratedImages/dzc_output_images/gjtccjesi160113_files/12/1_6.jpg]
